# Lying on the Dissection Table: Anatomizing Faked Responses

**DOI:** 10.3758/s13428-021-01770-8

**Published:** 2022-02-07

**Authors:** Jessica Röhner, Philipp Thoss, Astrid Schütz

**Affiliations:** Department of Psychology, Otto-Friedrich-Universität Bamberg, D-96045 Bamberg, Germany

**Keywords:** assessment, detection of faking, machine learning, self-report measures, Implicit Association Tests (IATs)

## Abstract

Research has shown that even experts cannot detect faking above chance, but recent studies have suggested that machine learning may help in this endeavor. However, faking differs between faking conditions, previous efforts have not taken these differences into account, and faking indices have yet to be integrated into such approaches. We reanalyzed seven data sets (*N* = 1,039) with various faking conditions (high and low scores, different constructs, naïve and informed faking, faking with and without practice, different measures [self-reports vs. implicit association tests; IATs]). We investigated the extent to which and how machine learning classifiers could detect faking under these conditions and compared different input data (response patterns, scores, faking indices) and different classifiers (logistic regression, random forest, XGBoost). We also explored the features that classifiers used for detection. Our results show that machine learning has the potential to detect faking, but detection success varies between conditions from chance levels to 100%. There were differences in detection (e.g., detecting low-score faking was better than detecting high-score faking). For self-reports, response patterns and scores were comparable with regard to faking detection, whereas for IATs, faking indices and response patterns were superior to scores. Logistic regression and random forest worked about equally well and outperformed XGBoost. In most cases, classifiers used more than one feature (faking occurred over different pathways), and the features varied in their relevance. Our research supports the assumption of different faking processes and explains why detecting faking is a complex endeavor.

Attempting to detect faking seems comparable to a pathologist’s work when attempting to clarify the cause of sudden death. Both endeavors are important and time-consuming and must take various circumstances into account. Indicators may depend on the circumstances under which the deed occurred (e.g., Röhner et al., 2013), an enormous pool of data must be evaluated to answer the question, and incorrect decisions can have severe consequences. And obviously, both efforts are based on the assumption that transgressors leave traces that will unveil them.

Recent research has suggested that people use different approaches when they fake on psychological measures (e.g., Bensch et al., 2019). Thus, they may also leave different traces. As faking is multifold, its detection is still a challenge, and even experts often fail to detect fakers above chance (e.g., Fiedler & Bluemke, 2005). In this study, we reanalyzed seven data sets by using machine learning to investigate whether artificial intelligence can help to detect faking when faking occurs under different conditions.

## Faking: An Unresolved Problem

In research and in applied settings, psychologists test hypotheses, explore behavior, and provide diagnoses. To do so, they typically have to rely on the sincerity of the people who participate in psychological assessments. Thus, an important quality criterion of psychological measures is their non-fakeability (e.g., Moosbrugger & Kelava, 2020). But an immense body of research has shown that people are able to fake on psychological measures (e.g., Birkeland et al., 2006; Viswesvaran & Ones, 1999). Even going beyond classical tests, measures that had originally been considered to be immune against faking (e.g., Implicit Association Tests; IATs; Greenwald et al., 1998) have turned out to be fakeable (e.g., Röhner et al., 2011; Röhner & Lai, 2021). As faking results in changes in test scores and rank orders, it is a serious problem that can impair the validity of tests (e.g., Salgado, 2016; see Ziegler et al., 2012, for an overview), and this impairment of validity may be higher for construct validity than for criterion validity (e.g., Ones & Viswesvaran, 1998; Ziegler & Buehner, 2009).

## Faking Detection as a Solution?

The goal of detecting faked scores in psychological measurement has been pursued for more than 100 years now (Sackett et al., 2017). A variety of approaches have been tested, including the implementation of scales that aim to measure the tendency to create favorable impressions (e.g., Paulhus, 2002) or the inspection of response latencies (e.g., Holden & Lambert, 2015). So far, though, none of these procedures has become widely accepted. Some procedures have been criticized for carrying their own risks (e.g., erroneously suspecting people high in conscientiousness to be fakers; Uziel, 2010, see also Röhner & Schütz, 2020). Others can only be applied to a very restricted group of measures (e.g., Röhner et al., 2013), or their applicability depends on measurement conditions (e.g., Röhner & Holden, 2021). Apparently, it is not as easy to detect faking as one might assume at first glance.

## What Makes Faking Detection a Challenge?

### The Complexity of Faking

Faking is affected by a complex interplay of conditions (e.g., Goffin & Boyd, 2009; Tett & Simonet, 2011; see also Röhner & Schütz, 2019) and can be pursued via different pathways (e.g., Bensch et al., 2019; Röhner et al., 2013). Faking detection is based on the idea that fakers leave telltale traces. However, if faking can be done in various ways and is impacted by conditions, faking detection is a complex endeavor in which different faking conditions have to be taken into account.

#### The Impact of Measures

Faking varies between measures (e.g., Röhner et al., 2011; Ziegler et al., 2007). For example, faking on self-reports includes decoding the items and choosing one’s responses according to the impression one wants to make (e.g., faking good vs. faking bad). By contrast, faking on IATs involves decoding the measurement procedure, which is based on reaction times (and error values [i.e., correct or erroneous responses]), and manipulating one’s reaction times (and error values) to achieve the desired impression (e.g., Röhner et al., 2013). Consequently, various theoretical approaches have suggested that faking on IATs is more difficult, and thus less possible, than faking on self-reports (see, e.g., De Houwer, 2006). In line with this argument, research has found more evidence of faking on self-reports than on IATs (e.g., Röhner et al., 2011; Steffens, 2004).

#### The Impact of Faking Direction

Several studies have demonstrated that faking depends on the requested faking direction (e.g., faking good vs. faking bad, Bensch et al., 2019; faking high scores vs. low scores, Röhner et al., 2013).^1^ Typically there is more evidence of faking when low scores are faked than when high scores are faked (e.g., Röhner et al., 2011; Viswesvaran & Ones, 1999).

#### The Impact of Knowledge

Faking depends on whether people have knowledge about measurement procedures and whether they are provided with strategies on how to fake (i.e., informed faking) or not (i.e., naïve faking; Röhner et al., 2013).^2^ It has been argued that informed faking improves people’s ability to fake (e.g., Raymark & Tafero, 2009; Snell et al., 1999). This idea has received empirical support (Röhner et al., 2011), and there was more evidence of faking when participants had prior information than when they were naïve (e.g., Röhner et al., 2013).

#### The Impact of Practice

Practice with faking on a specific measure can impact faking on that measure. There is more evidence of faking when participants are able to practice faking compared with when they are not (e.g., Röhner et al., 2011).

#### The Impact of Constructs

Research has indicated that faking also depends on the construct that fakers are attempting to fake. Differences in face validity have been shown to impact faking (Bornstein et al., 1994) and might explain why constructs that have more face validity than others are related to stronger faking behavior. Some studies have shown that the better participants can understand what is being measured, the more they are able to fake (e.g., McFarland & Ryan, 2000). However, the results of studies that have explored the impact of constructs have been less clear than the results of studies on other faking conditions. For example, Steffens (2004) demonstrated more faking on extraversion than on conscientiousness in IATs and self-reports, whereas Birkeland et al. (2006), who investigated only self-reports, demonstrated more faking on conscientiousness than on extraversion. However, the face validity of measures should not vary that strongly. Thus, this difference cannot be explained by face validity alone. Because there has been a lot of variation in other faking conditions that impact faking in these previous studies, it is not possible to ultimately explain such differences. Most likely, various constructs impact faking differently under different conditions. Therefore, the possibility that constructs impact faking should be considered.

To sum up, fakers will leave *different traces* under different faking conditions. When aiming to conduct research on faking detection, it is necessary to include the abovementioned conditions.

### Large Quantities of Data

Whereas the idea to investigate response patterns in order to identify faking goes back to Zickar et al. (2004), Calanna et al. (2020) recently showed that the use of response patterns (i.e., all of a participant’s responses; e.g., all answers to all items on a self-report) outperforms the use of scores (e.g., the test score from a self-report) in faking detection. Apparently, there is relevant information in response patterns that is not mirrored by scores (e.g., Kuncel & Borneman, 2007; Kuncel & Tellegen, 2009). Thus, to identify fakers, it seems necessary to compare various patterns of faked and not-faked responses. Consequently, large quantities of data have to be analyzed. Depending on the respective measure, data matrices quickly become very large (e.g., the IAT response pattern of a single participant includes about 250 reaction times and about 250 response values [i.e., erroneous or correct responses] that need to be compared with data from other participants).^3^ Considering the variety of faking behavior, a human analyst may be overburdened. And in fact, a study in which experts were asked to distinguish fakers from non-fakers on the basis of measurement protocols (i.e., response patterns) found that experts were unable to distinguish between these groups above chance (Fiedler & Bluemke, 2005).

To sum up, faking detection seems to work better when response patterns instead of scores are included. However, human analysts are typically overwhelmed by the amount of data related to analyzing response patterns.

### Faking Indices are not Available for all Measures

Faking indices seem to offer the ideal solution because they do not require researchers to investigate entire response patterns. Instead, researchers can inspect only certain indicators, thus making the analyses much more manageable. Usually, cutoff scores for these indices are suggested. When the indices miss the cutoffs, researchers can assume that participants have faked. Indices are typically based on theories about *how people fake* (e.g., Röhner et al., 2013). However, indices that have received empirical support are available for only a few measures (e.g., Cvencek et al., 2010; Röhner et al., 2013).

To sum up, efforts to detect faking have faced a kind of dead end. Inspecting response patterns is overwhelming for a human analyst and probably does not even lead to faking detection above chance levels—and although faking indices are more manageable, they are not yet available for all measures. Therefore, it makes sense to ask whether there might be another solution.

## Machine Learning as a Solution?

In recent years, machine learning has sparked immense interest and has been applied to several psychological problems (e.g., Calanna et al., 2020; Youyou et al., 2015). Machine learning may help solve the problem of complexity in faking detection. Artificial intelligence, in contrast to human analysts, can easily compare hundreds of responses on measures under different conditions, point to differences, and provide advice on how to detect faking. Thus, machine learning seems to be an ideal approach when the goal is to find out what fakers do and how their behavior differs from non-fakers (i.e., identifying the traces of faking; e.g., Calanna et al., 2020).

### The Process of Machine Learning

Classifiers are machine learning algorithms that classify objects (e.g., participants’ data) into groups (e.g., faker vs. non-faker). In principle, the goal of such classifiers is to use a chosen set of variables (i.e., features; e.g., response patterns, scores, or faking indices) to predict an outcome (i.e., faker vs. non-faker) on the basis of mathematical models (Kotsiantis et al., 2006). Supervised machine learning makes the classifier learn how to map observations (e.g., responses) onto categories (e.g., faker vs. non-faker) in a training process that is similar to human inductive reasoning (e.g., Xue & Zhu, 2009). In this process, the classifier is confronted with training data. The goal of the learning process is for the classifier to be able to correctly predict the categories (here, fakers and non-fakers) when it is confronted with new data. In a process of tuning, there is a search for the model that performs best while the settings of hyperparameters are adjusted. In the testing process, the classifier is applied to data that have not been part of the training data to validate the quality of the classification results (testing the generalizability of the classifier).

It is important to note that classifiers search for differences between the groups (e.g., fakers and non-fakers) in order to make the classifications. Thus, the stronger the difference in the behavior of fakers and non-fakers, the better the classifiers are at spotting the fakers.

### Performance Evaluation of Classifiers

The performance of classifiers is typically evaluated with the following performance indices (e.g., Calanna et al., 2020): *F1, Precision, Recall, Accuracy,* and the *Area Under the Curve (AUC)*. *F1* represents the harmonic mean of Precision and Recall.^4^*Precision* (or Positive Predictive Power) is the ratio of correctly classified positive observations (here, correctly identified fakers) to the number of observations labeled positive by the model (here, all participants who have been classified as fakers, including those who were non-fakers [i.e., false positives]). *Recall* (or Sensitivity) represents the ratio of correctly classified positive observations (here, correctly identified fakers) to the number of positive observations in the data (here, the number of fakers who were included in the data). *Accuracy* (or Efficiency) represents the ratio of observations that have been classified correctly (here, fakers as being fakers and non-fakers as being non-fakers) to the number of all observations in a given data set (here, fakers and non-fakers). The *AUC* is the Area Under the Curve in Receiver Operating Characteristic (ROC) curve analyses. In ROC curve analyses, hit rates (here, successfully identifying individuals as fakers) are plotted as a function of false-alarm rates (here, falsely identifying non-fakers as fakers; i.e., false negatives). The AUC shows the success rate of correct classifications (see also Röhner et al., 2013). It should be different from chance (i.e., .50) in a binary classification.

### Feature Importance

Exploring the importance of features (i.e., variables that are used to classify fakers from non-fakers here) allows researchers to peer into the black box of faking (e.g., Röhner & Ewers, 2016). Taking a look at the importance of the features offers insights into what (most) fakers did and whether their behavior varied across conditions.

## Status Quo Faking Detection With Machine Learning

### Machine Learning is Able to Detect Fakers

Boldt et al. (2018) used native Bayes, support vector machines, multinomial logistic regression, multilayer perceptron, simple logistic regression, propositional rule learner, and random forest on data from a self-developed IAT and showed that machine learning was able to detect fakers successfully. Machine learning performed better than Agosta et al.’s (2011) IAT faking index. A study by Calanna et al. (2020) used logistic regression, random forest, and XGBoost on data from a self-report measure (i.e., Big Five Questionnaire-2; BFQ2; Caprara et al., 2007). They found that machine learning was able to correctly classify fakers and non-fakers beyond a faking index (i.e., the lie scale from the BFQ2). However, neither study analyzed different faking conditions.

### Input Data Impact Classification Success

Calanna et al. (2020) varied their input data (i.e., response patterns vs. scores) and showed that response patterns led to better classification performances than scores. From a practical and theoretical point of view, the use of faking indices in combination with machine learning (i.e., as input data) seems to provide a meaningful extension for detecting faking because classifiers perform best when the input data are relevant for classification (e.g., Plonsky et al., 2019). Stated differently, using large quantities of data (e.g., response patterns) that are partly irrelevant for the classification problem (e.g., trials or items that are not faked at all) does not necessarily improve classification. However, focusing on relevant input data (e.g., validated indices) has the potential to outperform classification with response patterns and scores. Still, research has yet to test whether a combination of machine learning and faking indices may work better than using only response patterns or scores.

### The Quality of Detection Depends on Classifiers

Calanna et al. (2020) found that XGBoost worked best in faking detection. Boldt et al. (2018) showed that logistic regression worked best.^5^ Because these two studies differed with respect to measures, constructs, and faking directions, this difference may be explained by factors in the study designs. Still, both studies showed that the classifier impacts how well faking can be detected.

### Shortcomings and Open Questions

#### Impact of Faking Conditions

So far, research on the ability of machine learning to detect faking has not considered the complexity of faking under different faking conditions. *First*, faking depends on the measure (e.g., Röhner et al., 2011), and thus, a comparison between different measures seems essential. Previous research has focused on faking either an IAT (Boldt et al., 2018) or a self-report (Calanna et al., 2020), but results have not been compared between the two measures. Typically there is more evidence of faking on self-reports than on IATs, and thus, classifiers (which search for differences between fakers and non-fakers) should be superior at spotting fakers on self-reports than on IATs. *Second*, faking direction impacts faking (e.g., Bensch et al., 2019; Röhner et al., 2013). There is more faking of low scores than of high scores, and thus, classifiers should be better at detecting faked low scores than at detecting faked high scores. However, previous studies have either included only one faking direction (i.e., faking good; Calanna et al., 2020) or did not distinguish between faking directions (Boldt et al., 2018). *Third*, faking differs between naive and informed conditions (e.g., Röhner et al., 2013), and there is more evidence of faking when participants have information than when they are naïve (Röhner et al., 2011). Thus, it is plausible that faking detection is superior in informed than in naïve faking. However, Calanna et al. (2020) used naïve faking conditions, whereas Boldt et al. (2018) used only informed faking. *Fourth*, the impact of faking practice has not been taken into account. Thus, we do not know whether machine learning is able to detect both experienced fakers and novices. This distinction is important because one study indicated more evidence of faking with practice (Röhner et al., 2011), which in turn should somewhat increase its detection. *Fifth*, faking may depend on the constructs that are being faked (Steffens, 2004). So far, studies either did not discriminate systematically between constructs (Calanna et al., 2020) or used only one construct (Boldt et al., 2018). In order to show that a result can be generalized, different constructs have to be investigated and analyzed separately.

#### Implementation of Faking Indices

Both studies tested machine learning against faking indices but did not combine the two approaches by using these indices as input data. Given that classifiers perform best when input data are relevant for classification, research that includes empirically validated faking indices as input data is still needed.^6^

#### Peering Into the Black Box of Faking

The classification process has so far remained a black box because previous studies have not investigated the information the classifiers use to separate fakers from non-fakers under varying faking conditions. However, such an investigation is warranted to understand what makes fakers stand out.

## The Present Study

To advance knowledge about the ability of classifiers to detect faking, we built on research by Boldt et al. (2018) and Calanna et al. (2020) and reanalyzed seven data sets to address the abovementioned shortcomings. We compared two frequently used types of measures (self-reports vs. IATs). We included the faking of high scores and the faking of low scores. Although we focused on naïve faking attempts because they would provide the biggest challenge to the classifiers, we also included informed faking.^7^ We used data from participants with and without faking experience to investigate practice effects. We used data on four different constructs (extraversion, conscientiousness, need for cognition, and self-esteem). Concerning the IATs, we additionally took advantage of the benefits of having empirically supported faking indices by including them as input data. Finally but importantly, we investigated feature importance so that we could peer into the black box of faking. For reasons of comparison, we used the classifiers that turned out to be the best in Boldt et al.’s (2018) and Calanna et al.’s (2020) studies and those that had been used in both studies. Thus, we used logistic regression, random forest, and XGBoost as classifiers.

In doing so, we aimed to test the following hypotheses:

1. Considering that there is more evidence of faking on self-reports than on IATs, we expected classifiers to spot fakers better on self-reports than on IATs.
2. Considering that there is more evidence of faking when people fake low scores, we expected classifiers to spot faking low better than faking high.
3. Considering that there is more evidence of faking in informed conditions, we expected classifiers to spot informed faking better than naïve faking.
4. Considering that there is more evidence of faking after practice, we expected faking detection by classifiers to be superior when fakers are experienced than when they are not.
5. Considering that there might be differences in faking behavior with respect to constructs, we explored whether classifiers can detect faking to comparable extents across constructs (extraversion, conscientiousness, need for cognition, self-esteem).
6. Concerning self-reports, we tried to replicate the superiority of using response patterns over using scores as input data for machine learning in faking detection. For IATs, we tried to extend previous knowledge by showing that the use of empirically supported faking indices as input data in machine learning outperforms the use of response patterns and scores.
7. We wanted to replicate differences in faking detection with respect to types of classifiers.
8. We explored which kind of information classifiers use to detect faking under the varying conditions.

## Method

### Data

Altogether we used seven data sets (*N* = 1,039) that were collected from student samples under varying conditions: Data Set 1 comprised 84 participants (74 students; 64 women, 20 men; average age: 22.37 years, *SD* = 4.45), Data Set 2 comprised 197 participants (196 students, 1 no response; 165 women, 31 men, 1 diverse/no response; average age: 21.44 years, *SD* = 2.95), Data Set 3 comprised 260 participants (257 students; 191 women, 69 men, 3 diverse/no response; average age: 21.22 years, *SD* = 4.74), Data Set 4 comprised 293 participants (293 students; 220 women, 73 men; average age: 22.31 years, *SD* = 4.09), Data Set 5 comprised 199 participants (199 students; 163 women, 36 men; average age: 21.53 years, *SD* = 3.18), Data Set 6 comprised 299 participants (299 students; 225 women, 73 men, 1 diverse/no response; average age: 22.06 years, *SD* = 4.07), and Data Set 7 comprised 84 participants (74 students; 64 women, 20 men; average age: 22.37 years, *SD* = 4.45).

In each data set, participants worked on a baseline assessment and afterwards were randomly assigned to one of the following conditions: faking high scores, faking low scores, or working under the standard instructions of the measures (i.e., control condition). Whether they were asked to fake naïvely or whether they additionally received information about faking strategies varied between the studies (see Table 1). Also, whether they had faking practice varied between the studies (Table 1). In each data set, the constructs were assessed via IATs and self-reports, with the IATs always preceding the self-reports. When participants had missing values, we dropped those participants from the respective analyses.

**Table 1:** Means, Standard Deviations, and Reliabilities

Data set	Measurement occasion	Self-report			IAT		
		*M*	*SD*	α	*M*	*SD*	Split-half reliabilities
Extraversion							
1	Baseline	30.02	6.26	.75	0.24	0.44	.86
	Naive faking without practice	25.16	13.47	.94	0.16	0.57	.91
	Informed faking without practice	26.57	12.34	.95	0.19	0.86	.97
2	Baseline	29.77	6.27	.77	0.35	0.35	.73
	Naive faking without practice	26.41	13.68	.95	0.24	0.48	.81
	Naive faking with one practice trial	26.64	14.62	.96	0.20	0.47	.79
	Naive faking with two practice trials	27.06	14.77	.96	0.20	0.46	.70
	Naive faking with three practice trials	26.69	15.36	.97	0.20	0.47	.79
3	Baseline	28.00	6.26	.80	0.21	0.41	.84
	Naive faking without practice	26.03	14.34	.96	0.14	0.61	.88
4	Baseline	27.70	7.20	.85	0.12	0.43	.83
	Naive faking without practice	25.94	14.84	.97	0.13	0.56	.81
Conscientiousness							
5	Baseline	32.53	7.04	.86	0.56	0.30	.71
	Naive faking without practice	28.05	14.46	.97	0.45	0.43	.80
	Naive faking with one practice trial	27.73	15.65	.98	0.39	0.47	.80
	Naive faking with two practice trials	27.75	16.57	.98	0.37	0.44	.75
	Naive faking with three practice trials	27.39	16.59	.98	0.38	0.45	.79
Need for cognition							
6	Baseline	16.02	11.88	.87	-0.04	0.44	.78
	Naive faking without practice	5.76	31.76	.98	0.00	0.57	.84
Self-esteem							
7	Baseline	23.10	4.98	.87	0.70	0.28	.78
	Naive faking without practice	19.11	10.62	.98	0.47	0.48	.86
	Informed faking without practice	20.23	9.21	.97	0.30	0.93	.96
	Informed faking with one practice trial	20.01	9.54	.97	0.36	0.87	.93
	Informed faking with two practice trials	19.81	9.08	.97	0.37	0.78	.96

Descriptives for self-reports were based on questionnaire data with a possible range from 0 to 4 (extraversion), 0 to 4 (conscientiousness), -3 to +3 (need for cognition), or 0 to 3 (self-esteem). Descriptives for the IAT were based on IAT data, which were treated with the recommended *D*_2_ scoring algorithm (Greenwald et al., 2003a, 2003b). α was calculated as Cronbach’s α. Split-half reliability was based on split-half correlations incorporating Spearman-Brown adjustments.

#### Naïve Faking Without Faking Practice^8^

Naïve faking of high and low scores without practice was assessed for four constructs: extraversion (Data Set 1: Röhner et al., 2013; Data Set 2: Röhner, 2014a; Data Set 3: Allramseder, 2018; Dirk, 2017; Doukas, 2017; Hütten, 2018, Möller, 2017; and Data Set 4: Klink, 2017; Möller, 2017; Rudat, 2016), conscientiousness (Data Set 5: Röhner, 2014b), need for cognition (Data Set 6: Klink, 2017; Möller, 2017; Rudat, 2016), and self-esteem (Data Set 7: Röhner et al., 2011). In all of these studies, naïve faking followed the assessment of a baseline score obtained with the respective type of measure (i.e., IATs and self-reports).

#### Naïve Faking With Faking Practice^9^

Naïve faking of high and low scores with one, two, or three practice trials was assessed for two constructs: extraversion (Data Set 2: Röhner, 2014a) and conscientiousness (Data Set 5: Röhner, 2014b). In both data sets, naïve faking with one, two, or three practice trials followed a baseline assessment on the respective measure and the assessment of an initial naïve faking attempt without practice on the respective measure.

#### Informed Faking Without Faking Practice^10^

Informed faking of high and low scores without practice was assessed for two constructs: extraversion (Data Set 1: Röhner et al., 2013) and self-esteem (Data Set 7: Röhner et al., 2011). In both studies, informed faking without practice followed a baseline assessment on the respective measure and the assessment of an initial naïve faking attempt without practice in faking on the respective measure. Concerning Data Set 1, participants had to fake low if they had faked high under naïve faking conditions, and vice versa.

#### Informed Faking With Faking Practice^11^

Informed faking of high and low scores with one or two practice trials was assessed for self-esteem (Data Set 7: Röhner et al., 2011). Concerning informed faking with two practice trials, participants faked low if they had faked high under naïve faking conditions, and vice versa.

## Measures to be Faked

According to their randomly assigned experimental condition, participants were asked to fake either high or low scores or to work under standard instructions.

### Self-Reports

#### Extraversion Scale

Participants worked on the respective scale from the NEO-Five Factor Inventory (Borkenau & Ostendorf, 2008; English version: Costa Jr. & McCrae, 1992). This scale consists of 12 items that are answered on a 5-point rating scale ranging from 1 (*strongly disagree*) to 5 (*strongly agree*). Scale characteristics and Cronbach’s alpha reliability (Table 1) were comparable to Borkenau and Ostendorf’s (2008) values of *M* = 28.38, *SD* = 6.70, and α = .80.

#### Conscientiousness Scale

Participants worked on the respective scale from the NEO-Five Factor Inventory (Borkenau & Ostendorf, 2008; English version: Costa Jr. & McCrae, 1992). The scale consists of 12 items that are answered on a 5-point rating scale ranging from 1 (*strongly disagree*) to 5 (*strongly agree*). Scale characteristics and reliability (Table 1) were comparable to Borkenau and Ostendorf’s (2008) values of *M* = 30.87, *SD* = 7.13, and α = .84.

#### Need for Cognition Scale

Participants worked on the German adaptation of the 16-item short version of the need for cognition scale (Bless et al., 1994; English version: Cacioppo & Petty, 1982). The scale consists of 16 items that are answered on a 7-point scale ranging from -3 (*strongly disagree*) to +3 (*strongly agree*). Scale characteristics and reliability (Table 1) were comparable to Fleischhauer et al.’s (2010) values of *M* = 15.28, *SD* = 11.14, and α = .84.

#### Rosenberg Self-Esteem Scale

Participants worked on the German adaptation of the Rosenberg Self-Esteem Scale (von Collani & Herzberg, 2003; English version: Rosenberg, 1965). The scale consists of 10 items that are answered on a 4-point scale ranging from 0 (*strongly disagree*) to 3 (*strongly agree*). Scale characteristics and reliability (Table 1) were comparable to two data sets by von Collani and Herzberg’s (2003) of *M* = 22.67, *SD* = 4.81, and α = .84 and *M* = 22.73, *SD* = 4.95, and α = .85.

### IATs

The extraversion, conscientiousness, and self-esteem IATs consisted of seven blocks of trials. The single dimension Practice Blocks, 1, 2, and 5 each included 24 trials. The combined Blocks, 3, 4, 6, and 7 each consisted of 48 trials. The need for cognition IAT consisted of five blocks of trials (Fleischhauer et al., 2013). The single dimension Practice Blocks 1, 2, and 4 each included 22 trials (20 practice trials and two warm-up trials). The combined Blocks 3 and 5 each included 22 + 62 trials (20 practice trials and two warm-up trials; 60 experimental trials and 2 warm-up trials).

Between participants, IATs were counterbalanced for the order of combined phases^12^ to control for the effect that IAT scores tend to show stronger associations for the first pair of categories (Schnabel et al., 2008). Within participants, the presentation of combined phases was held constant. We used the R code provided by Röhner and Thoss (2019) to compute the *D*_2_ algorithm suggested by Greenwald et al. (2003a, 2003b) as a measure of the IAT effect. In addition, we calculated the diffusion-model-based IAT effect IAT_*v*_ (Klauer et al., 2007) by subtracting parameter *v* of the compatible phase from parameter *v* of the incompatible phase. For diffusion modeling, we followed the tutorial by Röhner and Thoss (2018) and used the EZ software, which can be downloaded (http://www.ejwagenmakers.com/papers.html).^13^

#### Extraversion IAT

This IAT (Back et al., 2009) included the target discrimination between self-relevant (e.g., I, mine) and non-self-relevant (e.g., they, their) words and attribute discrimination between extraversion-related words (e.g., talkative, active) and introversion-related words (e.g., shy, passive). The IAT’s characteristics (Table 1) were comparable to the values of *M* = 0.02, *SD* = 0.38, α = .85 reported by Back et al. (2009). Back et al. (2009) computed their mean with the *D*_1_ measure that does not involve a lower tail treatment, which explains why their mean was somewhat lower than ours because we used the recommended *D*_2_ measure (i.e., trials below 400 ms are deleted).

#### Conscientiousness IAT

This IAT (Steffens & Schulze König, 2006) included the target discrimination between self-relevant (e.g., I, mine) and non-self-relevant (e.g., they, their) words and attribute discrimination between conscientiousness-related words (e.g., strong-willed, pedantic) and nonconscientiousness-related words (e.g., aimless, laid-back). The IAT’s characteristics (Table 1) were comparable to the values of *M* = 0.53, *SD* = 0.28, and α = .81 reported by Steffens and Schulze-König (2006).

#### Need for Cognition IAT

This IAT (Fleischhauer et al., 2013) included the target discrimination between me (e.g., me, mine) and not me (e.g., they, others) words and the attribute discrimination between words related to reasoning (e.g., to scrutinize, to puzzle) and words related to relaxation (e.g., to chill, to daydream) attributes. The IAT’s characteristics (Table 1) were comparable to the values of *M* = -0.08, *SD* = 0.29, and α = .88 reported by Fleischhauer et al. (2013).

#### Self-Esteem IAT

This IAT (Greenwald & Farnham, 2000; Rudolph et al., 2006) included the target discrimination between self-relevant (e.g., I, mine) and non-self-relevant (e.g., they, their) words and the attribute discrimination between pleasant (e.g., joy, smile) and unpleasant words (e.g., disaster, war). The IAT’s characteristics (Table 1) were comparable to the values of *M* = 0.62, *SD* = 0.33, split-half-reliability = .85, *M* = 0.58, *SD* = 0.32, split-half-reliability = .83, and *M* = 0.64, *SD* = 0.30, split-half-reliability = .80, reported by Rudolph et al. (2008).

## Analytic Strategy

### Manipulation Check

We computed robust ANCOVAs (Wilcox, 2017) on each measure’s score to check whether participants in the faking groups were motivated and able to fake on all measures and whether their scores still differed when the baseline scores were controlled for (Vickers & Altman, 2001). As expected, the significant differences between trimmed means in nearly all design points revealed that participants in the faking conditions were motivated and able to fake on all measures. The results of the robust ANCOVAs are stored in the Supplement on the OSF (https://osf.io/bj492/). Moreover, faking led to typical consequences (e.g., Salgado, 2016); the means decreased, and the standard deviations and reliability scores increased (see Table 1).

### Computation of the Input Data

We used the data from the data sets described above and prepared the respective input data (i.e., response patterns, scores, and faking indices). *Response patterns* consisted either of all IAT trials (IATs) or of all item responses (self-reports).^14^*Scores* consisted of either *D*_2_ and IAT_*v*_ (IATs) or the test score (self-reports).^15^ We combined the potential of faking indices with the potential of machine learning by using faking indices as additional input data for classifiers. We based our set of faking indices on recommendations from prior research. We were unable to consider *faking indices* for self-reports because such validated indices are missing. Lie scales have come under heavy criticism (e.g., De Vries et al., 2014; Lanz et al., 2021; Uziel, 2010), and even the scale’s authors strongly advise against the use of lie scales to detect faking (e.g., Borkenau & Ostendorf, 2008). *Faking indices* for IATs were created on the basis of recommendations from prior research (see Agosta et al., 2011; Cvencek et al., 2010; Röhner et al., 2013; Röhner & Thoss, 2018). Accordingly, they consisted of CTS, IAT_*a*_, $\mathrm{IA}{\mathrm{T}}_{t_0}$, Ratio 150-10000, Slow_Co, and IncErr_Co for the naïve faking and informed faking of low scores. They consisted of CTS, IAT_*a*_, $\mathrm{IA}{\mathrm{T}}_{t_0}$_,_ Ratio 150-10000, and Accel_Co for the naïve faking of high scores and CTS, IAT_*a*_, $\mathrm{IA}{\mathrm{T}}_{t_0}$_,_ Ratio 150-10000, and Slow_In for the informed faking of high scores.^16^

### Computation of Faking Indices

#### Combined Task Slowing (CTS)

CTS was computed by subtracting the faster combined phase of the baseline IATs from the slower combined phase of the faked IATs (Cvencek et al., 2010). Therefore, average reaction times on the combined phases from the faked IATs were examined relative to the average reaction times on the combined phases from the baseline IATs.

#### ***IAT***_***a***_***and***$\boldsymbol{IA}{\boldsymbol{T}}_{{\boldsymbol{t}}_{\mathbf{0}}}$

Both indices were computed using the diffusion model analyses (e.g., Klauer et al., 2007; Röhner & Ewers, 2016) that we explained above. IAT_*a*_ represents participants’ speed-accuracy tradeoffs and was computed by subtracting parameter *a* of the compatible phase from parameter *a* of the incompatible phase, whereas $\mathrm{IA}{\mathrm{T}}_{t_0}$ represents participants’ non-decision-related processes and was computed by subtracting parameter *t*_0_ of the compatible phase from parameter *t*_0_ of the incompatible phase (Klauer et al., 2007).

#### Ratio 150–10000

This index was calculated according to the procedures described in Agosta et al. (2011). Thus, only reaction times between 150 and 10,000 ms were used, and the others were excluded from further analyses. Errors were substituted with the mean of the corresponding IAT phase with an added penalty of 600 ms. The average reaction times from the fastest combined phase (i.e., either compatible or incompatible) were then divided by the average reaction times from the corresponding single blocks (i.e., Single Blocks 1 & 2, or Single Blocks 1 & 5 for extraversion, conscientiousness, and self-esteem IATs; Single Blocks 1 & 2, or Single Blocks 1 & 4 for the need for cognition IAT).

#### Slow_Co, IncErr_Co, Slow_In, and Accel_Co

We computed these indices as described in Röhner et al. (2013). Thus, for the naïve as well as informed faking of *low scores*, we computed slowing down on the congruent phase (i.e., Slow_Co) as the difference in reaction times between the congruent IAT phase after faking instructions and the congruent IAT phase at baseline. For the naïve faking of low scores, we additionally computed increasing errors on the congruent phase (i.e., IncErr_Co) as the difference in errors between the congruent IAT phase under faking instructions and the congruent IAT phase at baseline. Albeit not necessarily related to faking success, this index was shown to mirror a faking strategy that is commonly used under the naïve faking of low scores.^17^ Concerning the naïve faking of high scores, we computed acceleration on the congruent phase (i.e., Accel_Co) as the difference in reaction times between the congruent IAT phase at baseline and the congruent IAT phase under faking. Concerning the informed faking of high scores, we computed slowing down on the incongruent phase (i.e., Slow_In) as the difference in reaction times between the incongruent IAT phase under faking and the incongruent IAT phase at baseline.

### Machine Learning

In order to investigate the ability of machine learning to detect faking, we used the following three types of classifiers on the faked and non-faked data: *logistic regression, random forest*, and *XGBoost*. We decided to use logistic regression and random forest for reasons of comparability. Both were used in Boldt et al. (2018) as well as in Calanna et al. (2020). We also included the classifier that worked best in each study: logistic regression (Boldt et al., 2018) and XGBoost (Calanna et al., 2020). Each of the classifiers was applied to *response patterns* and *scores* for the self-reports and to *response patterns*, *scores* and *faking indices* for the IAT. We thereby discriminated between the abovementioned faking conditions. Additionally, we made sure that the groups (i.e., faking and non-faking) were equal in size before we ran the analyses. A detailed overview of the resulting models is stored on the OSF (https://osf.io/bj492/).

Machine learning was performed with R (version 4.0.3) using the following packages: caret (version 6.0-86; Kuhn, 2020), ggh4x (version 0.1.0.9000; van den Brand, 2020), glmnet (version 4.0-2; Friedman et al., 2010), haven (2.4.3; Wickham & Miller, 2021), MLmetrics (version 1.1.1; Yan, 2016), pROC (version 1.16.2; Robin et al., 2011), R.utils (version 2.10.1; Bengtsson, 2020), randomForest (version 4.6-14; Liaw & Wiener, 2002), ROCR (version 1.0-11; Sing et al., 2005), tidyverse (version 1.3.0; Wickham et al., 2019), xgboost (version 1.2.0.1; Chen et al., 2020), and xlsx (version 0.6.4.2; Dragulescu & Arendt, 2020) and included training, tuning, and testing the classifiers as well as visually representing the results.

### Multilayer Cross-Validation

To ensure the generalizability of the results, we followed Calanna et al. (2020) and adopted a multilayer cross-validation procedure. We ran a five-fold cross-validation to tune the algorithms and additionally ran another 10-fold cross-validation to estimate their performance (see Cawley & Talbot, 2010). Training data and test data were independent from each other in every fold (i.e., data split). This was true for the five-fold cross-validation that was used to tune the algorithms and also for the 10-fold cross-validation that was used to estimate the performance.

### Performance Evaluation

When it comes to faking, *Precision* and *Recall* are equally important. Thus, we used the random search to find the best set of hyperparameters relative to the *F1* score in order to maximize the tradeoff between Precision and Recall (e.g., Calanna et al., 2020).

### Feature Importance

To gain insight into the black box of faking, we explored the features that were used by the classifiers to discriminate between fakers and non-fakers (see Fig. 4; for more details, see also Tables S7 to S9 and Figures S1 to S4 in the Supplement).

## Results

### Ability of Classifiers to Detect Fakers

**Fig. 1:**
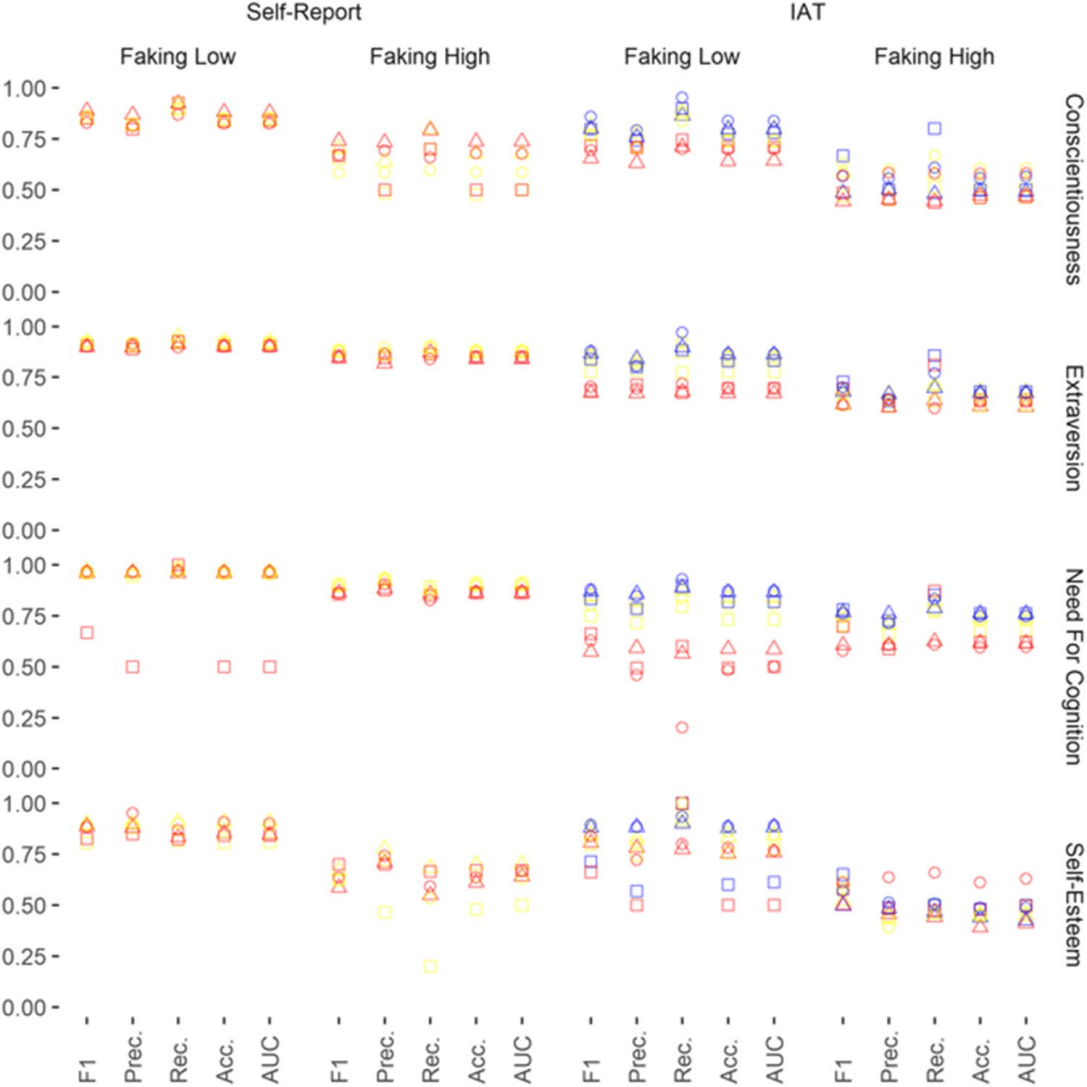
Performance Evaluation of the Classifiers: Naïve Faking Without Practice. *Note.* The five performance evaluation indices are presented on the x-axis. Prec. = Precision; Rec. = Recall; Acc. = Accuracy. Performance evaluation can vary between 0.00 and 1.00 (y-axis). Geometrical shapes code the classifiers: Circles represent performance evaluations from logistic regression, triangles represent performance evaluations from random forest, and squares represent performance evaluations from XGBoost. Colors code the kind of input data: Yellow represents response patterns, red represents scores, and blue represents faking indices

**Fig. 2:**
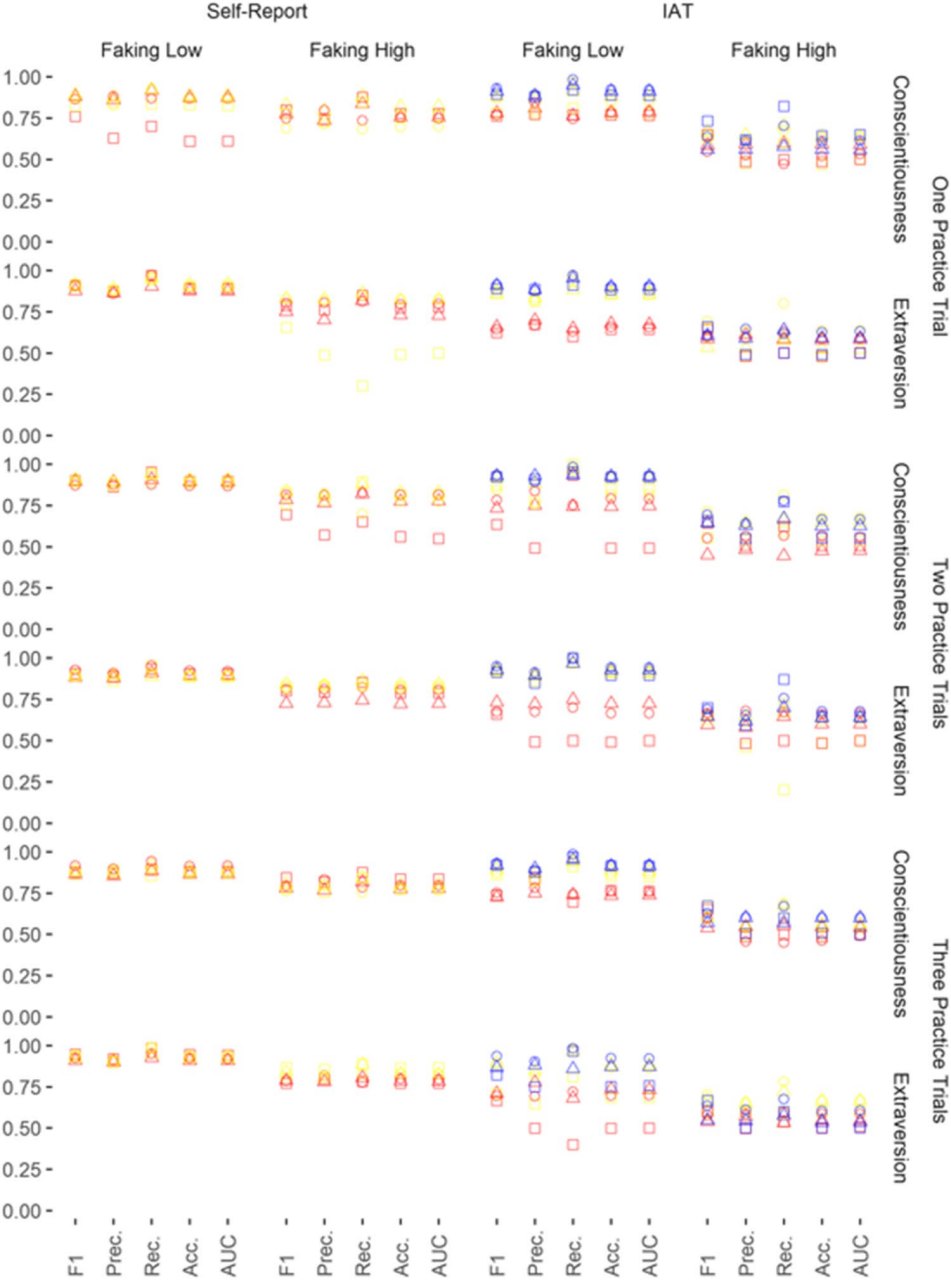
Performance Evaluation of the Classifiers: Naïve Faking With Practice. *Note.* The five performance evaluation indices are presented on the x-axis. Prec. = Precision; Rec. = Recall; Acc. = Accuracy. Performance evaluation can vary between 0.00 and 1.00 (y-axis). Geometrical shapes code the classifiers: Circles represent performance evaluations from logistic regression, triangles represent performance evaluations from random forest, and squares represent performance evaluations from XGBoost. Colors code the kind of input data: Yellow represents response patterns, red represents scores, and blue represents faking indices

**Fig. 3:**
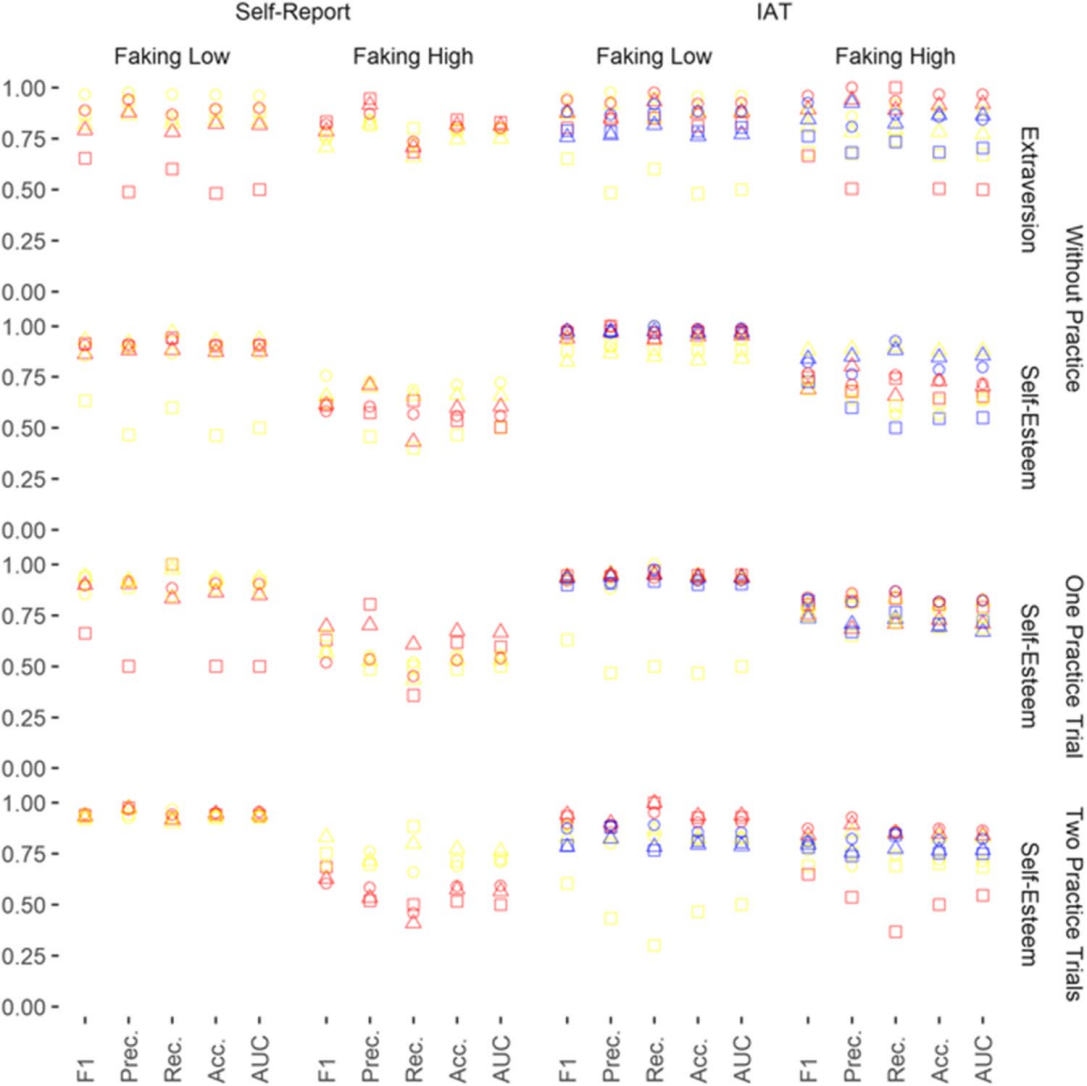
Performance Evaluation of the Classifiers: Informed Faking Without and With Practice. *Note.* The five performance evaluation indices are presented on the x-axis. Prec. = Precision; Rec. = Recall; Acc. = Accuracy. Performance evaluation can vary between 0.00 and 1.00 (y-axis). Geometrical shapes code the classifiers: Circles represent performance evaluations from logistic regression, triangles represent performance evaluations from random forest, and squares represent performance evaluations from XGBoost. Colors code the kind of input data: Yellow represents response patterns, red represents scores, and blue represents faking indices

Figures 1, 2 and 3 show performance evaluations of classifiers regarding the detection of faking under different faking conditions. A detailed overview of all performance evaluation indices is stored in the Supplement on the OSF (https://osf.io/bj492/).

To reduce complexity in the Results section, we evaluated performance by reporting the means and standard deviations of only the most important performance index with regard to faking detection (i.e., *F1*; the harmonic mean between Precision and Recall). Higher values on this performance index indicate better faking detection. In order to facilitate interpretation, we compared the *F1* performance evaluations using Cohen’s *d*.

Summing up, in most cases, the classifiers were able to detect faking above chance. As expected, however, faking conditions, input data, and type of classifier determined how well faking could be detected. *F1* varied from .44 (faking condition: naïve faking of high scores on the conscientiousness IAT without practice; classifier: random forest; input data: scores) to .98 (faking condition: informed faking of low scores on the self-esteem IAT without practice; classifier: logistic regression or random forest; input data: scores or indices).

We want to exemplify the results for these models. Concerning the model that was computed for the condition involving the naïve faking of high scores on the conscientiousness IAT without practice using the random forest classifier and scores as the input data, *F1* was .44. Precision was .45. Thus, only 45% of the participants who were classified as fakers actually were fakers (i.e., 55% were non-fakers). Recall was .44. Thus, only 44% of the fakers that existed were detected (i.e., 56% of the fakers were not detected). Accordingly, *F1* was below 50%. The probability of detecting fakers as fakers was below chance. Conversely, in the models that were computed for the condition involving the informed faking of low scores on the self-esteem IAT without practice and using the logistic regression or random forest classifier and scores or indices as input data, the chances of classifying fakers correctly as fakers were largely above chance. Concerning the model that was computed for the condition involving the informed faking of low scores on the self-esteem IAT without practice and using the logistic regression or random forest classifier and scores as input data, Precision was 1.00 (i.e., 100% of the participants who were classified as fakers actually were fakers. Thus, no non-fakers were classified as fakers), and Recall was .97 (97% of the fakers that existed were detected. Thus, only 3% of the fakers were missed). Concerning the model that was computed for the condition involving the informed faking of low scores on the self-esteem IAT without practice and using the logistic regression or random forest classifier and scores or indices as input data, Precision was .97 (97% of the participants who were classified as fakers actually were fakers; 3% were non-fakers that had been wrongly assigned to the group of fakers). Recall was 1.00 (100% of the fakers that existed were detected. No faker was missed).

### Self-Reports Versus IATs

The *F1* performance evaluations of classifiers were strongly superior on self-reports than on IATs when naïve faking without practice (*d* = -1.03, 95% CI [-1.42, -0.65]) and naïve faking with practice trials (*d* = -1.00, 95% CI [-1.32, -0.68]; Table 2; Figs. 1 and 2) had to be detected. Thus, classifiers were largely better at spotting fakers on self-reports than on IATs.

**Table 2:**
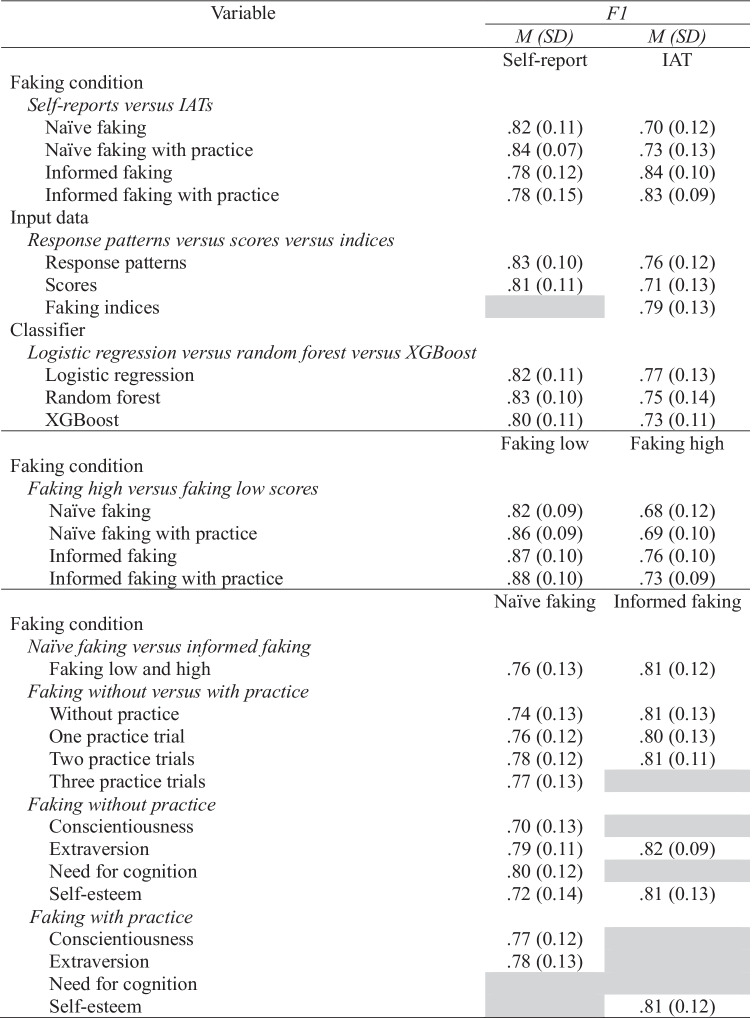
Means and Standard Deviations for the F1 Performance Measure

Grey cells indicate that these models were not part of our reanalyses because of the nonavailability of the recommended faking indices for self-reports or because we did not collect data concerning this condition.

Under informed faking conditions, the *F1* performance evaluations of classifiers on self-reports and IATs were more comparable than under naïve conditions. This was true concerning informed faking without practice (*d* = -0.55, 95% CI [-1.08, -0.03]) and with practice (*d* = -0.43, 95% CI [-0.95, 0.10]; Table 2; Fig. 3). Thus, differences in faking detection were less pronounced here.

### Faking High Versus Faking Low Scores

The *F1*performance evaluations of classifiers were strongly superior at detecting the faking of low scores as compared with high scores for naïve faking (*d* = -1.25, 95% CI [-1.64, -0.86]), naïve faking with practice trials (*d* = -1.79, 95% CI [-2.13, -1.44]), informed faking (*d* = -1.10, 95% CI [-1.64, -0.56]), and informed faking with practice trials (*d* = -1.58, 95% CI [-2.16, -1.00]; see Table 2; Figs. 1, 2 and 3). Thus, faking was spotted much better for the faking of low scores than for the faking of high scores.

### Naïve Faking Versus Informed Faking

The *F1* performance evaluations of classifiers were somewhat better under informed faking than under naïve faking (*d* = -0.40, 95% CI [-0.61, -0.18]; see Table 2; Figs. 1 and 3). Thus, informed faking was spotted somewhat better than naïve faking.

### Faking Without Versus with Practice

The *F1* performance evaluations of classifiers were comparable between experienced and inexperienced fakers for naïve faking without practice versus one practice trial (*d* = -0.08, 95% CI [-0.39, 0.23]), without practice versus two practice trials (*d* = -.24, 95% CI [-0.55, 0.07]), and without practice versus three practice trials (*d* = -0.15, 95% CI [-0.46, 0.16]; Table 2; Fig. 1) as well as for informed faking without practice versus one practice trial (*d* = -0.08, 95% CI [-0.58, 0.43]) and without practice versus two practice trials (*d* = 0.00, 95% CI [-0.51, 0.51]; (Table 2; Fig. 3). Thus, classifiers worked equally well irrespective of faking practice.

### Conscientiousness Versus Extraversion Versus Need for Cognition Versus Self-Esteem

When naive faking without practice had to be detected, the *F1* performance evaluations of classifiers were comparable regarding the constructs need for cognition and extraversion (*d* = -0.09, 95% CI [-0.59, 0.42]; Table 2; Fig. 1). They were somewhat superior for detecting faking on need for cognition and extraversion versus detecting faking on self-esteem: need for cognition versus self-esteem (*d* = -0.61, 95% CI [-1.12, -0.10]), extraversion versus self-esteem (*d* = -0.56, 95% CI [-1.07, -0.04]). They were strongly superior for detecting faking on need for cognition and extraversion versus detecting faking on conscientiousness: need for cognition versus conscientiousness (*d* = -0.80, 95% CI [-1.33, -0.27]), extraversion versus conscientiousness (*d* = 0.75, 95% CI [-1.27, -0.22]; Table 2; Fig. 1).

When aiming to detect the naïve faking of participants with practice trials, *F1* performance evaluations of classifiers were comparable for the constructs extraversion and conscientiousness (*d* = -0.08, 95% CI [-0.37, 0.21]; Table 2; Fig. 2). Also, when aiming to detect informed faking, *F1* performance evaluations of classifiers were comparable for the constructs extraversion and self-esteem (*d* = -0.09, 95% CI [-0.60, 0.42]; Table 2; Fig. 3). Thus, classifiers were comparably good at detecting fakers on different constructs when participants had practice or information.

### Response Patterns Versus Scores Versus Indices

*F1* performance evaluations of classifiers were comparable when response patterns and scores were used to detect faking on self-reports (*d* = -0.19, 95% CI [-0.49, 0.11]; Table 2; Figs. 1, 2 and 3).

Concerning IATs, *F1* performance evaluations of classifiers demonstrated that the use of faking indices outperformed the use of response patterns, which in turn outperformed the use of scores: faking indices versus response patterns (*d* = -0.24, 95% CI [-0.54, 0.06]), response patterns versus scores (*d* = -0.40, 95% CI [-0.71, -0.09]), and faking indices versus scores (*d* = -0.62, 95% CI [-0.93, -0.31]; Table 2)

### Logistic Regression Versus Random Forest Versus XGBoost

*F1* performance evaluations of classifiers were comparable when logistic regression was used to detect faking and when random forest was used to detect faking (*d* = -0.08, 95% CI [-0.31, 0.16]; Table 2). Logistic regression significantly outperformed the use of XGBoost, but random forest did not: logistic regression versus XGBoost (*d* = -0.25, 95% CI [-0.48, -0.01]) and random forest versus XGBoost (*d* = -0.17, 95% CI [-0.40, 0.07]; Table 2).

## Opening the Black Box: Which Information did Classifiers Use to Detect Faking?

Because logistic regressions worked best to detect faking, we decided to focus on analyses of the feature importance of logistic regressions in order to reduce complexity. Also, we decided to focus on the feature importance of faking indices in IATs and response patterns in self-reports because, overall, these approaches were the most successful for detecting faking.

**Fig. 4:**
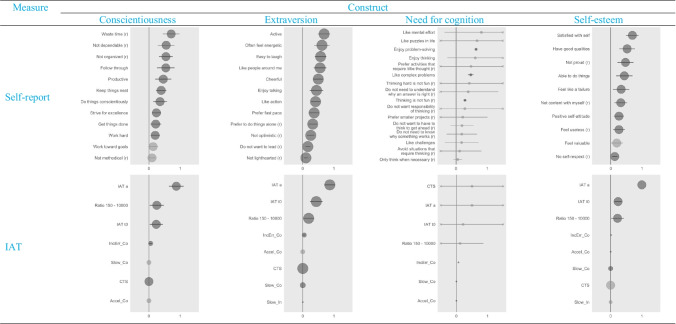
Forest Plots of the Evaluation of Feature Importance in Logistic Regression. *Note.* The x-axis represents the mean feature importance, which can vary between 0 = not important at all to 1 = most important. The larger the distance from zero, the more important the feature is. Point size is proportional to the number of occurrences (*N*) used to calculate the mean feature importance and can vary on the basis of the underlying data or the results of the algorithm that was used. Horizontal lines represent confidence intervals. Confidence intervals that exceeded the margins of -0.5 and 1.5 were clipped. Clipping is indicated by an “x.” Confidence intervals that fall below zero are colored in a lighter shade of grey, or else they are blue. Response patterns represent the features of self-reports. Faking indices represent the features in IATs

Figure 4 provides an overview of the aggregated feature importance of logistic regressions in the form of forest plots (see Figure S4 in the Supplement for the plots from random forest and XGBoost). It clearly demonstrates that, for IATs, participants’ speed-accuracy setting (i.e., IAT_*a*_) was consistently the most important feature for detecting faking,^18^ whereas the results on the response pattern in self-reports were more diverse. Although there was clear variation within feature importance, the differences between the relevance of various items was less strongly pronounced. On this general level, the most important feature on the extraversion scale represents *activity,* the one concerning the conscientiousness scale represents *handling of time*, the one concerning the need for cognition represents *enjoyment of problem-solving*, and the one on the self-esteem scale represents *self-satisfaction.*

Thus, additional analyses of feature importance on a more detailed level (i.e., with respect to faking conditions) seemed relevant. Figures S1 to S3 in the Supplement show the feature importance of classifiers under the different faking conditions. Feature importance clearly demonstrates that faking occurs along different pathways, which is why we decided to present the most important feature and compare the ordering of feature importance with Spearman’s rank correlation coefficients. Tables S6 to S8 in the Supplement provide an overview of the *M* and *SD* values for features between fakers and non-fakers. To assess correspondence between feature importance under different faking conditions, we calculated Spearman’s rank correlation coefficients between the ranked (descriptive) importance of features under different faking conditions with each other (1 = most important; 6 to 16^19^ = least important).

### Self-Reports

#### Conscientiousness Scale

Under naïve faking conditions without practice, the most important feature for detecting the faking of low scores was the lower ratings of fakers on Item 8 (i.e., “When I make a commitment, I can always be counted on to follow through”), whereas the most important feature for detecting the faking of high scores was the higher ratings of fakers on Item 6 (i.e., “I waste a lot of time before settling down to work”; Table S7). The same was true for faking after one or two practice trials (Table S8). It was different when faking with three practice trials: The most important feature for detecting the faking of low scores was the lower ratings of fakers on Item 10 (i.e., “I am a productive person who always gets the job done”), and the most important feature for detecting the faking of high scores was the higher ratings of fakers on Item 11 (i.e., “I never seem to be able to get organized”; Table S8).

The ordering of feature importance varied with respect to faking direction. The importance of features for detecting the faking of low scores was always unrelated to the importance for detecting the faking of high scores under naïve faking without practice (*r*_*s*_ = -.13, *p* = .697) as well as with one (*r*_*s*_ = -.20, *p* = .542), two (*r*_*s*_ = .08, *p* = .795), or three (*r*_*s*_ = .50, *p* = .095) practice trials. Feature importance orderings also varied with respect to practice. The features that had the strongest impact on detecting fakers under naïve faking conditions without practice did not have the strongest impact on detecting fakers under naïve faking conditions with one practice trial for faking low (*r*_*s*_ = .40, *p* = .191) or faking high (*r*_*s*_ = .24, *p* = .475) or two practice trials for faking low (*r*_*s*_ = .46, *p* = .131) or faking high (*r*_*s*_ = .55, *p* = .067). But the results for naïve faking held for three practice trials for faking high (*r*_*s*_ = .68, *p* = .015) but not for faking low (*r*_*s*_ = .10, *p* = .762).

#### Extraversion Scale

Under naïve faking conditions without practice, the most important feature was the lower ratings of fakers on Item 2 (i.e., “I laugh easily”) when detecting the faking of low scores and the higher ratings of fakers on Item 7 (i.e., “I often feel as if I'm bursting with energy”) when detecting the faking of high scores (Table S7). With one or two practice trials in faking, the most important feature for detecting faked low scores was the lower ratings of fakers on Item 11 (i.e., “I am a very active person”), whereas the most important feature for detecting faked high scores again was the higher ratings of fakers on Item 7 (Table S8). When participants had three practice trials, the most important feature for detecting faked low scores was the lower ratings of fakers on Item 4 (i.e., “I really enjoy talking to people”), whereas the most important feature for detecting faked high scores was the higher ratings of fakers on Item 1 (i.e., “I like to have a lot of people around me”; Table S8). Under informed faking conditions, the most important feature was the lower ratings of fakers on Item 1 when detecting the faking of low scores and the higher ratings of fakers on Item 11 when detecting the faking of high scores (Table S9).

The ordering of feature importance for detecting the faking of low scores under naïve faking was not or only scarcely related to the detection of the faking of high scores without practice (*r*_*s*_ = .26, *p* = .417) or with one (*r*_*s*_ = .15, *p* = .649), two (*r*_*s*_ = .64, *p* = .026), or three practice trials (*r*_*s*_ = .25, *p* = .443) under naïve faking as well as under informed faking (*r*_*s*_ = .46, *p* = .131). Features varied with respect to practice concerning faking high but not concerning faking low. Features that had the strongest impact on detecting fakers of high scores under naïve faking conditions without practice also had the strongest impact on detecting fakers of high scores under naïve faking conditions with one practice trial (*r*_*s*_ = .60, *p* = .039), two practice trails (*r*_*s*_ = .82, *p* = .001), and three practice trials (*r*_*s*_ = .67, *p* = .017). Features that had the strongest impact on detecting fakers of low scores under naïve faking conditions without practice did not have the strongest impact on detecting fakers of low scores under naïve faking conditions with one practice trial (*r*_*s*_ = .51, *p* = .090), two practice trials (*r*_*s*_ = .04, *p* = .914), or three practice trials (*r*_*s*_ = .40, *p* = .199). Additionally, features varied with respect to whether participants faked naïvely or were informed about faking strategies for faking low (*r*_*s*_ = .11, *p* = .729) and faking high (*r*_*s*_ = .50, *p* = .101).

#### Need for Cognition Scale

Under naïve faking conditions without practice, the most important feature was the higher^20^ ratings of fakers on Item 3 (i.e., “I tend to set goals that can be accomplished only by expanding considerable mental effort”) when detecting the faking of low scores and the higher ratings of fakers on Item 13 (i.e., “I prefer my life to be filled with puzzles that I must solve”) when detecting the faking of high scores (Table S7). Again, features varied with respect to faking direction (*r*_s_ = .37, *p* = .154).

#### Self-Esteem Scale

Under naïve faking conditions without practice, the most important feature was the lower ratings of fakers on Item 3 (i.e., “I feel that I have a number of good qualities”) when detecting the faking of low scores and the higher ratings of fakers on Item 1 (i.e., “On the whole, I am satisfied with myself”) when detecting the faking of high scores (Table S7). Under informed faking conditions without practice, the most important feature was the lower ratings of fakers on Item 4 (i.e., “I am able to do things as well as most other people”) when detecting the faking of low scores and the higher ratings of fakers on Item 1 when detecting the faking of high scores (Table S9). With one practice trial, the most important feature was the higher^21^ ratings of fakers on Item 5 (i.e., “I feel I do not have much to be proud of”) when detecting the faking of low scores and the lower^22^ ratings of fakers on Item 9 (i.e., “All in all, I am inclined to think that I am a failure”) when detecting the faking of high scores (Table S9). With two practice trials, the most important feature was the lower ratings of fakers on Item 4 when detecting the faking of low scores and the higher ratings of fakers on Item 5 when detecting the faking of high scores (Table S9). Again, the ordering of feature importance for detecting the faking of low scores was unrelated to the ordering for detecting the faking of high scores for naïve faking (*r*_s_ = .03, *p* = .934), informed faking without practice (*r*_s_ = -.08, *p* = .829), informed faking with one practice trial (*r*_s_ = -.07, *p* = .855), and informed faking with two practice trials (*r*_s_ = .24, *p* = .511). Additionally, feature importance did not largely vary with respect to whether participants faked naïvely or were informed about how to fake when faking low (*r*_s_ = .69, *p* = .029), but it did vary when faking high (*r*_s_ = .33, *p* = .347). Finally, under informed faking, features varied with respect to practice when faking low (*r*_s_ = .42, *p* = .229) and when faking high (*r*_s_ = .29, *p =* .425).

### IATs

#### Conscientiousness IAT

Concerning the detection of faking under naïve faking conditions without practice, the most important feature was the lower IAT_a_ of fakers (i.e., participants’ speed-accuracy setting) when faking low scores and the lower Ratio 150-10000 of fakers (i.e., the ratio that measures a slowing down behavior on either the compatible or incompatible IAT phase compared with the single blocks) when faking high scores (Table S7). With practice in faking, the lower IAT_a_ of fakers was the most important feature for detecting the faking of low scores, and the higher IAT_a_ of fakers was the most important feature for detecting the faking of high scores (Table S8). The ordering of feature importance varied with respect to faking direction. Under naïve faking without and with practice, the ordering of feature importance for detecting the faking of low scores was not related to the ordering of feature importance for detecting the faking of high scores without practice (*r*_s_ = .54, *p =*.258), with one practice trial (*r*_s_ = .69, *p =* .060), with two practice trials (*r*_s_ = .30, *p =* .479), or with three practice trails (*r*_s_ = .69, *p =* .060). The ordering of feature importance did not vary greatly with respect to practice. Features that had the strongest impact on the detection of fakers under naïve faking conditions without practice also had the strongest impact on the detection of fakers under naïve faking conditions with one practice trial for faking low (*r*_s_ = .93, *p =* .001) and faking high (*r*_s_ = .73, *p =* .042), for two practice trails for faking low (*r*_s_ = .98, *p* ≤ .001) and faking high (*r*_s_ = .55, *p =* .158), and for three practice trails for faking low (*r*_s_ = .95, *p* ≤ .001) and faking high (*r*_s_ = .75, *p =*.032).

#### Extraversion IAT

Concerning the detection of faking under naïve faking without practice, the most important feature was the lower IAT_a_ of fakers when detecting the faking of low scores and the higher IAT_a_ of fakers when detecting the faking of high scores (Table S7). With practice in faking, a lower $\mathrm{IA}{\mathrm{T}}_{t_0}$ (one practice trial), a lower IAT_a_ (two practice trials), and a higher Ratio 150-10000 (three practice trials) of fakers were most important for detecting the faking of low scores, but a higher IAT_a_ (one and two practice trials) and a lower IAT_a_ (three practice trials) of fakers was consistently important for detecting the faking of high scores. Under informed faking conditions, the most important feature was the lower IAT_a_ of fakers when detecting the faking of low scores and the higher IAT_a_ of fakers when detecting the faking of high scores. Differences with respect to faking direction were also apparent on the extraversion IAT. Although under naïve faking without practice, the ordering of the importance of features for detecting the faking of low scores was somewhat related to the detection of the faking of high scores without practice, *r*_*s*_ = .76, *p* = .028, it was not related when participants had practice with one (*r*_*s*_ = .23, *p* = .578), or three practice trials (*r*_*s*_ = .46, *p* = .244), but with two practice trials (*r*_*s*_ = .79, *p* = .021). Under informed faking conditions, the ordering of the importance of features for detecting the faking of low scores was strongly related to the ordering for detecting the faking of high scores without practice, *r*_s_= .90, *p* = .002.

The ordering of the importance of features did not vary much with respect to practice. The features that had the strongest impact on detecting fakers under naïve faking conditions without practice also had the strongest impact on detecting fakers under naïve faking conditions with one practice trial for faking low (*r*_*s*_ = .93, *p* = .001) and faking high (*r*_*s*_ = .93, *p* = .001), two practice trails for faking low (*r*_*s*_ = 1.00, *p* ≤ .001) and faking high (*r*_*s*_ = .98, *p* ≤ .001), and three practice trails for faking low (*r*_*s*_ = .81, *p* = .015) and faking high (*r*_*s*_ = .98, *p* ≤ .001).

Additionally, feature importance did not vary much with respect to whether participants faked naïvely or whether they were informed for faking low (*r*_*s*_ = .86, *p* = .006) and faking high (*r*_*s*_ = .88, *p* = .004).

#### Need for Cognition IAT

Under naïve faking conditions without practice, the most important feature was the lower IAT_a_ of fakers when detecting the faking of low scores and the higher CTS (i.e., combined task slowing) of fakers when detecting the faking of high scores (Table S7). Again, features varied with respect to faking direction, *r*_s_ = .09, *p* = .840.

#### Self-esteem IAT

Concerning faking detection under naïve faking conditions without practice, the most important feature was the lower IAT_a_ of fakers when detecting the faking of low and high scores (Table S7). Under informed faking conditions without and with practice, the most important feature was also the lower IAT_a_ of fakers when detecting the faking of low scores and the higher IAT_a_ of fakers when detecting the faking of high scores (Table S9). The ordering of the importance of features varied with respect to faking direction. Under naïve faking, feature importance differed regarding the detection of low and high scores, *r*_s_ = .61, *p* = .106. As was true for extraversion, the orderings of the importance of features for high and low scores were more strongly related under informed faking (*r*_s_ = .80, *p* = .017), informed faking with one practice trial (*r*_s_ = .75, *p* = .032), and informed faking with two practice trials (*r*_s_ = .78, *p* = .024). Feature importance did not largely vary with respect to whether participants faked naïvely or whether they were informed about faking strategies for faking low (*r*_s_ =.71, *p* = .048) or faking high (*r*_s_ =.90, .002). Finally, under informed faking, feature orderings did not vary with respect to practice for faking low (*r*_s_ =.98, *p* ≤ .001) or faking high (*r*_s_ =1.00, *p* ≤ .001).

## Discussion

We reanalyzed seven data sets (*N* =1,039) to investigate the ability of machine learning to detect faking under different faking conditions. We analyzed the detection of faking on two frequently used and well-established psychological measures (self-reports and IATs) regarding the faking of high and low scores, naïve and informed faking, faking with and without practice, and on four constructs (extraversion, conscientiousness, need for cognition, and self-esteem), thus varying factors that have been shown to impact faking behavior (i.e., traces of faking). We also compared three types of classifiers (logistic regression, random forest, and XGBoost) and three types of input data (response patterns, scores, and faking indices). Last but not least, to peer into the black box of faking and its detection, we explored feature importance.

Our results are in line with Boldt et al.’s (2018) and Calanna et al.’s (2020) earlier findings, which identified machine learning as a promising approach for detecting faking. In most cases, classifiers were able to detect faking above chance. Our results extend previous findings by showing that besides the type of classifier and besides the type of input data, the conditions under which faking occurs affect how faking is done and how well it can be detected. Accordingly, faking detection ranged from chance levels to nearly 100%. For example, detection was rather poor with naïve faking on the conscientiousness IAT when using scores and random forest, but it worked very well for detecting the informed faking of low scores on the self-esteem IAT on the basis of scores or faking indices with logistic regression.

### Faking Detection is Better on Self-Reports than on IATs Under Naïve Conditions but not Under Informed Conditions

Under naïve faking and irrespective of practice levels, classifiers had more trouble recognizing fakers on IATs than on self-reports. Under informed faking, the opposite was true, albeit this effect was much smaller and nonsignificant when people had practice.^23^

Various theorizing has suggested that faking on IATs is more difficult and thus less possible than faking on self-reports (see, e.g., De Houwer, 2006). This argument has been supported by empirical research (e.g., Röhner et al., 2011; Steffens, 2004). In fact, the reduced transparency of the measurement procedure in IATs as compared with self-reports is one core attribute of IATs (e.g., De Houwer, 2006). Consequently, especially naïve faking conditions challenge participants when they try to fake, whereas information makes faking easier (e.g., Röhner et al., 2011). One explanation for this finding comes from research that shows that participants develop and use successful but also unsuccessful faking strategies in naïve faking conditions (Röhner et al., 2013). By contrast, faking on self-reports is quite easy because participants basically choose responses that fit the impression they want to make. Correspondingly, research has shown that faking on self-reports is not impacted much by knowledge about faking strategies (Röhner et al., 2011). Most likely, successful faking strategies are very obvious on self-reports, and thus, any potential gains from information about how to fake is less pronounced than it is on IATs.

Thus, the measure to be faked plays a role in faking detection. As expected, faking detection was better on self-reports than on IATs. However, keeping in mind the results on feature importance, this better detection on self-reports came at the expense of a lower generalizability of features to detect faking across faking conditions on the self-report measures than on the IATs. Moreover, this advantage of self-reports was only true for naïve faking. Thus, the impact of the type of measure on faking detection changes with information about faking strategies. Faking on less transparent measures (e.g., on IATs) was detected to almost the same degree as on self-reports when participants had information about how to fake them.

### The Detection of Faking Low is Superior to the Detection of Faking High

Earlier findings have emphasized that faking behavior differs by faking direction (e.g., Bensch et al., 2019; Röhner et al., 2013) and found more evidence of faking when participants faked low scores than when they faked high scores (see, e.g., Röhner et al., 2011). Extending these results and in line with expectations, classifiers were better at detecting faking low than at detecting faking high.

Thus, faking direction played a role in the detection of faking in the current study. Faked low scores were spotted better than faked high scores.

### The Detection of Informed Fakers is Superior to the Detection of Naïve Fakers

Previous research has found more evidence of faking when participants were informed than when they were naïve with respect to faking strategies—as informed faking is easier and thus more pronounced than naïve faking (e.g., Röhner et al., 2011). In line with this idea and as expected, classifiers performed somewhat better for informed faking than for naïve faking. Thus, although faking detection was possible for fakers who faked naïvely and those who were informed about how to fake, knowledge about faking strategies impacted faking detection; it was superior when participants had knowledge about faking strategies than when they did not.

### Practice in Faking has no Impact on Detection

Faking detection was equally good regardless of practice levels. Apparently, information (see paragraph above) is more relevant than practice.

### Without Practice and Without Information, Faking Detection is Better on Need for Cognition and on Extraversion Than on Self-Esteem and Conscientiousness

When participants faked naively and had no practice, the construct to be faked played a role. Detection was better for extraversion and need for cognition than for self-esteem and conscientiousness. These findings are in line with a finding by Lukoff (2012), who gave warnings to potential fakers and found that constructs impacted how well fakers and non-fakers were classified with machine learning.

However, when participants in our studies had practice in faking or were informed about faking strategies, detection did not differ between constructs. Apparently, faking became more homogeneous under these conditions.

To sum up, although it was possible to detect faking for all four constructs, the construct that was being faked impacted faking detection for conditions involving naïve faking without practice. Faking was more often detected when it involved extraversion or need for cognition than self-esteem or conscientiousness in this case.

### Faking Detection With Faking Indices as Input Data is Superior to Faking Detection With Response Patterns or Scores

Replicating Calanna et al.’s (2020) prior findings, our study demonstrated that faking detection is superior when using response patterns than when using scores as input data. These results are in line with the assumption that faking is represented more strongly in a kind of profile (response patterns) rather than in scores (Geiger et al., 2018). Apparently, faking is too multifaceted to be captured by one overall score (e.g., Röhner et al., 2013). The findings also underscore the advantage of machine learning in faking detection: Machines can analyze complex response patterns efficiently. However, whereas the effect for IATs was significant, it remained nonsignificant for self-reports. Most likely the quantity of response patterns plays an important role with respect to *whether* response patterns perform better than scores. In our analyses, response patterns on IATs consisted of 220 to 264 responses, whereas response patterns on self-reports consisted of 10 to 16 responses. The self-report measure used by Calanna et al. (2020) included 134 responses. Thus, the advantage of using response patterns seems especially strong for measures with large sets of responses. An obvious explanation for this may be that with more items, faking can be more multifaceted, and it becomes more important to inspect response patterns.

Extending these findings, we demonstrated that using response patterns can be outperformed when using theoretically derived and empirically supported faking indices—at least for IATs where such indices are available. This is in line with our expectation and can be explained by the fact that machine learning performs best if the input data are all relevant for classification. Thus, focusing on relevant input data only (e.g., indices that reflect empirically supported faking strategies) works better than including all IAT trials on which participants do not fake on all.

### Faking Detection With Logistic Regression and Random Forest is Superior to XGBoost

Whereas Calanna et al. (2020) showed that faking detection with XGBoost was superior to faking detection with random forest and logistic regression, Boldt et al. (2018) demonstrated that logistic regression worked best. In combining the detection of faking on self-reports and IATs, our research showed that in general, logistic regression and random forest worked comparably well, and logistic regression outperformed XGBoost. Calanna et al. (2020) focused on faking on a self-report and on faking high scores only, whereas Boldt et al. (2018) restricted their research to faking on an IAT. Thus, faking conditions most likely impact the performance of classifiers and thereby have to be taken into consideration when choosing which classifiers to use to detect faking.

Moreover, the level of measurement of input variables (continuous vs. categorical) may impact the performance of different machine learning algorithms. For instance, in many cases, logistic regression works better with continuous predictors (i.e., response patterns, scores, and faking indices in IATs as well as scores in self-reports) than with categorical predictors (i.e., response patterns in self-reports), whereas one strength of random forest is that its performance is excellent with categorical predictors. Thus, the level of measurement of input variables should also be taken into consideration when choosing potential machine learning algorithms.

### Which Behavior Revealed Fakers?

Exploring the importance of features provides insight into the processes of faking and in its detection. On a general level, for IATs, participants’ speed-accuracy setting (i.e., IAT_*a*_) was consistently the most important feature for detecting faking, whereas the results on the response pattern in self-reports were more diverse. On self-reports, self-descriptions concerning activity (extraversion), handling of time (conscientiousness), enjoyment of problem-solving (need for cognition), and self-satisfaction (self-esteem) were the most important for revealing faking on a general level, but there was much variation between faking conditions. Thus, overall, there was considerably more correspondence across IATs than across self-report measures, which especially supports the generalizability of findings for the detection of faking with faking indices on the IAT. Nevertheless, to a certain extent, our results allow for a look into the black box of faking processes in self-report measures. So far*,* there is little theoretical background to explain why some items strongly discriminated between fakers and non-fakers, whereas others were less important. However, research using a cognitive interview technique revealed that people evaluated the importance of an item in terms of the situational demand (e.g., Ziegler, 2011). If participants judge an item as important with regard to the situation, they will attempt to fake on that item—but they will not attempt to fake on items they regard as unimportant regarding their faking goal. According to Ziegler (2011), people use specific knowledge and implicit theories about the desired impressions to evaluate item importance. Further, the stakes of the situation may impact the evaluation of what is important (Ziegler, 2011). In our studies, for example, participants were confronted with a personal selection scenario, which most likely triggered specific knowledge and implicit theories about the characteristics of an ideal employee (e.g., Klehe et al., 2012). In our studies, the ideal employee on a general level may be described as someone who is active, does not waste time, enjoys problem-solving, and is happy with themselves. Still, there were differences with respect to faking conditions, and thus, there were no front-runners in feature importance across conditions. All in all, there is some evidence that, depending on the respective faking conditions, people consider different items to be relevant and thereby fake on different items. In addition, the following insights were indicated by more fine-grained analyses of feature importance.

First, the classifier used more than one feature (i.e., more than one faking index on IATs or more than one item on self-reports, respectively) to distinguish fakers from non-fakers.^24^ This finding is in line with the assumption that faking occurs through several pathways (Bensch et al., 2019; Röhner & Schütz, 2019).^25^ At maximum, all features were used (i.e., six features on IATs, up to 16 features on self-reports) for classification. Second, feature importance varied with respect to faking conditions. This finding shows that faking differs between conditions and that faking is consequently detected on the basis of different behaviors. The feature that had the largest impact on the classification varied with respect to faking direction. Concerning self-reports, different items (features) were considered to be most important for classification when detecting the faking of low scores and when detecting the faking of high scores. Also, the rank-orderings of features typically differed between the faking of high and low scores. With IATs, the strategy to adapt speed-accuracy tradeoffs was most important for both faking directions. This finding is in line with previous research that demonstrated that faking impacts the extent to which participants prioritize accuracy or speed in decision-making (Röhner & Lai, 2021; Röhner & Thoss, 2018). As in self-reports, the rank-orderings of features typically differed between the faking of high and low scores, except for informed faking. In other words, faking on IATs becomes more uniform with information. Thus, in line with previous theorizing (e.g., Bensch et al., 2019; Röhner & Schütz, 2019), two different processes appear to be behind the faking of high versus low scores. However, how different these processes are depends on the type of measure. Besides the differences with respect to faking direction, the rank-orderings of the importance of features also varied with respect to practice trials. On self-reports, practice in faking impacted the way participants responded to items when faking low scores in a naïve manner, but its impact was smaller when they faked high scores. By contrast, variation in the ordering of feature importance concerning the IAT was low: IAT participants used very similar faking strategies irrespective of practice levels. Last but not least, the ordering of the importance of features varied with respect to whether participants faked naively or in an informed manner on self-reports but not on IATs. Thus, informed fakers were detected on the basis of other features than naïve ones on self-reports, but on IATs, the features were similar between the two.

To sum up, feature importance analyses underpin prior theories that faking processes differ (e.g., Bensch et al., 2019). However, not only do they shed light on the question of how people fake under different faking conditions, but they also show that faking detection—in line with different faking behavior—occurs along very different pathways. Nevertheless, especially with regard to the self-report measures, correspondence across conditions is limited. Moreover, the statistical power differed between conditions. Thus, the generalizability of these results is a relevant issue for future research.

### Limitations

We considered a large quantity of variables that impact faking and its detection in order to advance knowledge about faking and its detection with machine learning. Nevertheless, our study is limited in that our data came only from participants who were instructed to fake. However, we purposefully did not include data from applied settings. Not only does instructing participants to fake represent the most common methodology that is used to investigate faking (Smith & McDaniel, 2012), but it also provides valuable insights into the extent to which people can fake and into the strategies people apply when asked to fake (Smith & Ellingson, 2002; Smith & McDaniel, 2012). This was what we were interested in and what we needed for our analyses. If the motivation to fake in applied settings would have been the focus of our research, we would have preferred to use data from applied settings. So, on the one hand, the data fit our research goal. On the other hand, there is one even more important reason for not including data from applied settings. In applied settings, participants are usually not instructed to fake, which creates a circular problem if researchers want to investigate the detection of faking. To classify fakers and non-fakers, one has to know first who was trying to fake, and this is exactly what the research is trying to find out. Instead of applying other faking indices that bear their own risks of misclassification, we decided to restrict ourselves to using instructed faking sets. Although faking has been suggested to be the sum of at least two substantive sources of variance (i.e., traits and faking; e.g., Bensch et al., 2019; Ziegler et al., 2015), variance shared across multiple traits could still be affected by various response sets and response styles. Thus, in applied settings without experimental manipulations, faking is not the only type of response distortion that occurs. To avoid this problem, we chose laboratory settings and experimentally manipulated faking by explicitly asking participants to fake in order to minimize the activation and impact of other response sets and response styles that might cloud the results (e.g., acquiescence, midpoint or extreme point responding, carelessness). Thus, future research should investigate whether the analytical procedures tested here can be generalized to other response sets and response styles.

Faking strategies can also differ between settings (e.g., applied settings vs. laboratory settings). On the one hand, it seems plausible that they are more diverse in applied than in laboratory settings (e.g., because of more diverse test-taker characteristics that stipulate more diverse faking strategies). On the other hand, even the contrary might be the case. Faking strategies could be less diverse in applied settings because of certain information, such as one prominent test-cracking manual or training that recommends one “most successful” faking strategy. These factors most likely impact the success of detecting faking with machine learning. Future research should investigate whether the procedures applied here can be generalized to real-world faking.

Furthermore, the machine learning approach that we applied in our study is based on the assumption that faking can be considered a dichotomous variable with two categories (i.e., faking and non-faking). This reasoning is supported by previous research that has demonstrated that faking can be grouped into distinct latent classes (Zickar et al., 2004) and is also in line with previous procedures that aimed to detect faking with machine learning (e.g., Calanna et al., 2020). However, there is also evidence that faking could be considered a continuous variable (i.e., it can be measured at any level of precision; Geiger et al., 2021; Geiger et al., 2018; Ziegler et al., 2015). Using dichotomous variables to predict continuous variables can result in information loss, and thus, in nonoptimal findings. Future research should therefore compare the results of attempts to measure faking as a dichotomous versus a continuous variable.

Also, we restricted ourselves exclusively to using faking indices that have already been empirically validated in past research and thus wanted to avoid intermingling potential concerns about the validity of faking indices with the validity of the machine learning approach. The applied indices differ in their meaning and limitations. Slow_Co, IncErr_Co, Slow_In, and Accel_Co have been theoretically derived and empirically shown to indicate faking. However, they can be used only when data are available from both a baseline and a faking condition, which researchers do not always have at hand. The same is true for CTS, which in addition is a bit difficult to interpret as it confounds a substantial IAT effect (i.e., a difference between compatible and incompatible effects; here, between different IATs) and a possibly superimposed faking strategy (e.g., intentional slowing in an IAT phase). By contrast, Ratio 150–10000 can be applied without participants’ baseline data. In addition, it is a very intuitive index of relative slowing on the compatible or incompatible phase relative to the preceding single blocks. Not only have IAT_*a*_ and $\mathrm{IA}{\mathrm{T}}_{t_0}$ been shown to be related to faking, but both indices additionally (and in contrast to the other indices) also correspond with a theoretical model (the drift diffusion model; e.g., Klauer et al., 2007). However, not only do they represent faking, but they also reflect substantial differences. For example, IAT_*a*_ reflects differences in participants’ perceptions of task difficulty, and $\mathrm{IA}{\mathrm{T}}_{t_0}$ reflects interferences during the selection of responses (Schmitz & Voss, 2012). Thus, in contrast to other indices, IAT_*a*_ and $\mathrm{IA}{\mathrm{T}}_{t_0}$ should not be interpreted as pure faking indices. Future research might evaluate additional experimental indices (e.g., standard distribution of reaction times) and compare them against indices that have already been empirically validated with machine learning.

Interestingly, feature importance was more consistent in IATs than in self-reports. Self-reports lack empirically validated faking indices, but such indices were used in our analyses on faking in IATs and performed best there. Thus, feature importance was most likely more consistent for IATs than for self-reports because the input data for IATs (faking indices), as compared with those for self-reports (response patterns) were superior in predicting faking. In combination with varying sample sizes, this might explain the differences between IATs and self-reports. The small amounts of data in certain conditions do not warrant tests of generalizability on the basis of multiple independent data sets, which might be a relevant extension of future research. Nevertheless, the results emphasize that a machine learning approach works best when input data are relevant for classification (e.g., Plonsky et al., 2019) as is the case with validated faking indices. By contrast, using large amounts of data (e.g., response patterns) that are partly irrelevant for the classification problem (e.g., trials or items that are not faked at all) does not necessarily improve classification. Instead, focusing on relevant input data (e.g., validated indices) has the potential to outperform classification with response patterns and scores.

### In a Nutshell: Can Machine Learning Assist in Faking Detection?

Under naïve faking, the detection of faking was superior on self-reports than on IATs, whereas this was not the case under informed faking. Thus, the type of measure plays a role, and nontransparent measurement procedures lead to lower success in faking detection, but this effect disappears with practice or information. In general, faking detection was superior for the faking of low scores compared with the faking of high scores. This finding is in line with prior theorizing that faking low and high represent different processes. This assumption is also backed up by feature importance analyses because the features that can be used to detect the faking of low scores typically differed from the ones that can be used to detect the faking of high scores.^26^ Faking detection was also superior for informed as compared with naïve faking. Thus, the good news is that test-cracking manuals might aid the detection of fakers because naïve faking is less homogenous and, thus, more difficult to detect. Fakers could be spotted comparably well regardless of their practice levels. Thus, information about how to fake is more relevant than practice in faking. Similarly, whereas the choice of construct impacted faking detection under naïve faking, it did not under informed faking or when participants had practice. Also, fakers were spotted best by machine learning with empirically validated faking indices or response patterns and worst by the use of scores—especially when there were long response patterns. Last but not least, the machine learning algorithm affected the quality of faking detection. As a consequence of the interplay of these conditions, faking detection varied from chance levels to 100%.

## Conclusion

Faking detection indeed resembles the work of a pathologist. By carefully anatomizing faked responses, our results showed that faking conditions largely impact faking behavior and thereby affect the quality of faking detection with machine learning. Additionally, faking behavior is reflected in different input data, which then impact the quality of faking detection. Moreover, the type of machine learning algorithms impact the quality of faking detection. Our analyses provided insights into faking processes and can explain why faking detection is such a complex endeavor. Not only do fakers fake on different pathways when confronted with different faking conditions, but in most cases, more than one pathway is used for faking. Thus, it is challenging to find typical traces left by fakers, thus rendering faking detection with machine learning a promising approach. However, a variety of factors that impact how well (from chance levels to excellent) machine learning works in faking detection has to be taken into consideration in this endeavor.

## Footnotes

1 A distinction typically used in personality assessment is faking good (trying to make a good impression) and faking bad (trying to make a bad impression), whereas the faking of high and low scores (referring to the scores that are faked) is a more typical distinction in research contexts (e.g., Röhner & Schütz, 2019).

2 As most people may have limited access to psychological measurement procedures, naïve faking is probably the predominant form of faking.

3 The exact number of IAT trials can differ from IAT to IAT. However, usually about 250 trials are included.

4 Because *Precision* and *Recall* are equally relevant when it comes to faking detection, *F1* is considered to be the most relevant index of performance here. Therefore, we focused on *F1* in the Results section, but all performance indices are available on the OSF and are also plotted in the figures.

5 For reasons of comparability, we refer to the unpruned data set from Boldt et al. (2018) here.

6 In addition, the majority of the data in the study by Calanna et al. (2020) were retrieved from a repository of real-world assessments that had been conducted prior to their study. Thus, although experimental manipulations could in principle also be conducted in naturalistic settings, Calanna et al. (2020) did not experimentally manipulate faking. Instead, they used a post hoc strategy and defined participants as fakers or non-fakers on the basis of their scores on a lie scale (faking index). However, the validity of such scales for identifying fakers has been criticized (e.g., De Vries et al., 2014; Goffin & Christiansen, 2003; Uziel, 2010). A recent meta-analysis by Lanz et al. (2021) revealed that scales that are intended to measure socially desirable responding are not suitable for measuring response biases (e.g., faking). Consequently, whether the assignment of fakers and non-fakers was valid is not clear for the majority of data in the study by Calanna et al. (2020), and thus, there is a need for an investigation based on experimentally manipulated faking attempts.

7 Informed fakers must follow a small set of faking strategies that strongly limit their behavior and are thereby “eye-catching” for classifiers. Thus, we focused on data from participants who were given freedom in *how* they faked low or high scores because this provided the more critical test for the classifiers.

8 Naive faking without practice refers to conditions in which participants had no prior practice in faking on these measures at all.

9 Naive faking with faking practice refers to conditions in which participants had one to three practice trials in faking naively on these measures.

10 Informed faking without practice refers to conditions in which participants had no prior practice in informed faking on these measures but had an initial naive faking attempt.

11 Informed faking with practice refers to conditions in which participants had one or two practice trials in informed faking on these measures and had an initial naive faking attempt.

12 We use the term combined phase to refer to the combination of the critical practice block and the critical test block (compatible phase = compatible practice trials and compatible test trials; incompatible phase = incompatible practice trials and incompatible test trials; see, e.g., Röhner & Ewers, 2016).

13 We followed Voss and Voss’ (2008) and Voss et al.’s (2013) recommendation to exclude outliers from the individual response-time distribution for participants who had reaction times lower than 200 ms or higher than 5,000 ms. Altogether, we removed 11,201 trials (2.02% of the trials). We removed 141 trials from the IAT (0.4% of the trials) in Data Set 1, 4,850 trials from the IAT (3.2% of the trials) in Data Set 2, 377 trials from the IAT (0.8% of the trials) in Data Set 3,626 trials from the IAT (1.1% of the trials) in Data Set 4, 4,054 trials from the IAT (2.7% of the trials) in Data Set 5, 1,017 trials from the IAT (2.1% of the trials) in Data Set 6, and 136 trials from the IAT (0.2% of the trials) in Data Set 7. As suggested by Wagenmakers et al. (2007), we corrected the percentage of correct responses that equaled exactly 1.0 by subtracting half an error from the percentage of correct responses before running further analyses. We also corrected the percentage of correct responses that equaled exactly 0 and 0.5 by adding half an error, respectively. Because of the approximation formula, *t*_0_ can be negative in sign (e.g., the mean of the reaction time is less than the mean decision time that is defined: $\frac{\mathrm{a}}{2\mathrm{v}}\times \frac{1-{\mathrm{e}}^{\mathrm{y}}}{1\mp {\mathrm{e}}^{\mathrm{y}}}$; Wagenmakers et al., 2007). However, a negative *t*_0_ cannot be interpreted theoretically because it represents the nondecisional portion of the response time, and time cannot take on negative values (Voss et al., 2004). Thus, participants with negative *t*_0_ should be removed before further analyses (Wagenmakers et al., 2007). Altogether, we excluded *N* = 68 (4.80% of participants) from further analyses because *t*_0_ was negative in sign (*N* = 4 [4.8%] participants from Data Set 1, *N* = 22 [11.2%] participants from Data Set 2, *N* = 5 [1.9%] participants from Data Set 3, *N* = 11 [3.8%] participants from Data Set 4, *N* = 14 [7.0%] participants from Data Set 5, *N* = 9 [3.0%] participants from Data Set 6, and *N* = 3 [3.6%] participants from Data Set 7). With fakers, there were *N* = 52 [3.7%] *t*_0_-based exclusions, whereas there were only *N* = 16 [1.1%] *t*_0_-based exclusions with non-fakers, indicating that faking attempts had a strong impact on reaction time distributions so that the mean decision time exceeded the mean reaction time, and thus, t_*0*_ was impacted (see also Röhner & Thoss, 2018). We used EZ to estimate independent diffusion models for each participant and each combined IAT phase type within every measurement occasion. Altogether, we computed 5,880 EZ diffusion models (Data Set 1: 336; Data Set 2: 1,576; Data Set 3: 520; Data Set 4: 586; Data Set 5: 1,592; Data Set 6: 598; and Data Set 7: 672. After we removed the outliers from each IAT, we obtained the following results. For the conscientiousness IAT, each diffusion model analysis was based on an average of 93.5 trials. For extraversion, each diffusion model analysis was based on an average of 94.0 trials. For self-esteem, each diffusion model analysis was based on an average of 95.8 trials. For need for cognition, each diffusion model analysis was based on an average of 78.3 trials.

14 Concerning the response patterns, in Data Set 3, we excluded the response patterns from the self-reports of two participants (i.e., 0.07% of all response patterns in self-reports) from machine learning because both participants had one missing response.

15 Concerning the scores, in Data Set 2, we excluded the IAT scores of five participants from the models that investigated naïve faking without practice in naïve faking, of four participants from the models that investigated naïve faking with one practice trial in naïve faking, of four participants from the models that investigated naïve faking with two practice trials in naïve faking, and of five participants from the models that investigated naïve faking with three practice trials in naïve faking because they had missing values on the respective measurement occasion. Concerning Data Set 5, we excluded the IAT scores of two participants from the models that investigated naïve faking without practice in naïve faking, of four participants from the models that investigated naïve faking with two practice trials in naïve faking, and of three participants from the models that investigated naïve faking with three practice trials in naïve faking because they had missing values on the respective measurement occasion. Concerning Data Set 6, we excluded the IAT scores of six participants from the models that investigated naïve faking without practice in naïve faking because they had missing values on the respective measurement occasion. Thus, a total of 1.12% of a total of 2,940 IAT scores were excluded.

16 Concerning the indices, we excluded the indices from participants in IATs when they had negative *t*0 parameters (Wagenmakers et al., 2007; see also Röhner & Lai, 2020). In Data Set 1, three participants were excluded concerning naïve faking without practice in naïve faking, and two participants were excluded concerning informed faking without practice in informed faking. In Data Set 2, five participants were excluded concerning naïve faking without practice in naïve faking, three participants were excluded concerning naïve faking with one practice trial in naïve faking, nine participants were excluded concerning naïve faking with two practice trials in naïve faking, and 12 were excluded concerning naïve faking with three practice trials in naïve faking. In Data Set 3, five participants were excluded concerning naïve faking without practice in naïve faking. In Data Set 4, 11 participants were excluded concerning naïve faking without practice in naïve faking. In Data Set 5, three participants were excluded concerning naïve faking without practice in naïve faking, four participants were excluded concerning naïve faking with one practice trial in naïve faking, five participants were excluded concerning naïve faking with two practice trials in naïve faking, and six participants were excluded concerning naïve faking with three practice trials in naïve faking. In Data Set 6, nine participants were excluded concerning naïve faking without practice in naïve faking. In Data Set 7, three participants were excluded concerning naïve faking without practice in naïve faking, one participant was excluded concerning informed faking with one practice trial in informed faking, and one participant was excluded concerning informed faking with two practice trials in informed faking. Thus, a total of 2.62% of a total of 2,940 IAT indices were excluded.

17 Actually, from a practical stance, all indications that might differ between faking and non-faking may be relevant because nonsuccessful fakers also nevertheless attempted to distort their responses. This is why we included this index.

18 Note that concerning need for cognition, the most important feature was the IAT_a_ score when low scores were faked and the CTS when high scores were faked. Thus, the two indices are equally important in Figure 4.

19 The number of the least important rank depended on the measure and condition. It ranged from 5 when faking high on the IAT to 16 when faking on the need for cognition scale.

20 Note that the item is inverted and was reinverted before the analyses here.

21 Note that the item is inverted and was reinverted before the analyses here.

22 Note that the item is inverted and was reinverted before the analyses here.

23 Under informed faking conditions, participants in the faking conditions on IATs were given strategies that involved the manipulation of responses on compatible and incompatible IAT phases, whereas they were told that they could fake the self-report by changing their responses on the items.

24 Concerning self-reports, the minimum number of features was two.

25 Note that if classifiers could identify all fakers with one feature, this would not imply that all fakers adopted the same strategy because each index and each item response can reflect diverse mechanisms and also diverse strategies.

26 The exception is that IAT_*a*_ is the most important feature for detecting the faking of high and low scores in most cases.
